# Dynamic Trends and Underlying Factors of COVID-19 Vaccine Booster Hesitancy in Adults: Cross-Sectional Observational Study

**DOI:** 10.2196/44822

**Published:** 2023-08-01

**Authors:** Jian Wu, Mingze Ma, Quanman Li, Xinghong Guo, Clifford Silver Tarimo, Shiyu Jia, Xue Zhou, Meiyun Wang, Jianqin Gu, Yudong Miao, Beizhu Ye

**Affiliations:** 1 Department of Health Management College of Public Health Zhengzhou University Zhengzhou, Henan China; 2 Henan Province Engineering Research Center of Health Economy & Health Technology Assessment Zhengzhou China; 3 Department of Science and Laboratory Technology Dar es Salaam Institute of Technology Dar es Salaam United Republic of Tanzania; 4 Department of Public Utilities Management College of Health Management Mudanjiang Medical University Hei Longjiang China; 5 Henan Provincial People's Hospital Zhengzhou University Zhengzhou China; 6 School of Medicine Southern University of Science and Technology Shenzhen China

**Keywords:** COVID-19 vaccine, vaccine hesitancy, COVID-19 booster vaccination, influencing factors, China

## Abstract

**Background:**

COVID-19 vaccine hesitancy reduces vaccination rates, which is detrimental to building herd immunity and halting the spread of COVID-19 and its variations. Most researches have simply identified the reasons affecting COVID-19 vaccination reluctance without delving into its dynamics, which makes forecasting future trends difficult.

**Objective:**

This study aimed to examine the current COVID-19 vaccine booster hesitancy rate in Chinese adults as well as the dynamics of vaccine hesitancy and its influencing factors. The results of this study will have practical implications for policy responses in mainland China, and effective COVID-19 booster vaccination in specific populations.

**Methods:**

The web-based survey was completed by creating questionnaires and using a stratified random sampling method to collect information from adults (≥18 years old) among 2556 households in 4 geographical regions of China. We collected sociodemographic information, health status, awareness of COVID-19 and its vaccine, self-perceptions, trust in medical staff and vaccine developers, and so on. The odds ratios and 95% CI for the statistical associations were estimated using logistic regression models.

**Results:**

Overall, 6659 participants (females: n=3540, 53.2%; males: n=3119, 46.8%) responded. In total, 533 (8%; 95% CI 7.4%-8.7%) participants presented a clear hesitancy in receiving the COVID-19 booster vaccination, while 736 (11.1%; 95% CI 10.3%-11.8%) expressed hesitancy in regular booster vaccination. A higher prevalence of vaccine hesitancy in both booster vaccination and regular booster vaccination was observed among participants with a history of allergies, experiencing chronic disease, lower levels of public health prevention measures or susceptibility or benefits or self-efficiency, higher levels of severity or barriers, and lower trust in both medical staff and vaccine developers (*P*<.05). The females and participants with higher education levels, higher levels of barriers, lower levels of susceptibility, and lower trust in vaccine developers preferred to have attitudinal changes from acceptance to hesitancy, while people with higher education levels, lower self-report health conditions, experiencing chronic disease, history of allergies, and lower trust in medical staff and developers were all positively associated with constant COVID-19 booster hesitancy.

**Conclusions:**

The prevalence of COVID-19 vaccine booster hesitancy is not high in mainland China. However, there is a slight increment in hesitancy on regular booster vaccination. Conducting targeted information guidance for people with higher education levels and chronic diseases, as well as improving accessibility to booster vaccination and increasing trust in medical staff and vaccine producers may be highly effective in reducing vaccine hesitancy.

## Introduction

Vaccinating a sufficient proportion of the population against COVID-19 is one of the most effective methods for achieving “herd immunity,” lowering morbidity, and increasing population survival. It is also the most cost-effective and straightforward intervention to prevent the COVID-19 pandemic [1,2]. Prevention of the COVID-19 virus and its variants through COVID-19 vaccination can confer benefits related to morbidity and mortality [3], mainly including the protection of immunocompromised populations such as infants, young children, and the elderly [4,5]. According to the latest reports from the World Health Organization (WHO), two-thirds of the world’s population is vaccinated, including 75% of health workers and older people. However, there are still wide disparities in vaccination rates [6]. The COVID-19 booster vaccination is an important tool for consolidating herd immunity and responding to the COVID-19 pandemic and deaths [7], and receiving the COVID-19 booster vaccine is essential to achieve adequate immunization coverage to control the global pandemic [8,9]. Understanding people’s attitudes to COVID-19 booster vaccination aids in developing COVID-19 booster vaccination plans, which ensure the consolidation of herd immunity and minimize health inequalities.

Since the global outbreak of the COVID-19 epidemic, several vaccines have been developed, such as inactivated vaccines, live-attenuated vaccines adenovirus vector vaccines, and recombinant protein vaccines [10,11]. Although these multiple vaccines are considered safe and effective [3,4], and countries around the world are increasing the supply of vaccines, waiving vaccination fees [12], and actively promoting the COVID-19 booster vaccination, there are still concerns about vaccine hesitancy, which affects the vaccination rates of COVID-19 vaccines as well as booster vaccination. This situation may not be helpful to bolster herd immunity in order to prevent the further spread of COVID-19 and its variations. According to the WHO, “Vaccine hesitancy” has been considered as one of the top 10 public health problems across the world [13-15]. It refers to the delay or refusal to receive safe vaccination services, which can accompany the use of vaccines [3,15-17]. The danger of vaccine hesitancy is that it can lead to immunization of populations below the herd immunity threshold and ultimately fail to protect the populations. This is particularly true for people whose immunity is very weak to be vaccinated, such as infants, the elderly, or patients with serious illnesses, who are the first to be exposed to infectious diseases if the herd immunity barrier is breached [4,18]. The COVID-19 vaccine booster hesitancy is a type of vaccine hesitancy and is a continuation of people’s hesitancy about the COVID-19 vaccine. Therefore, what factors influence people’s hesitancy to receive the COVID-19 booster vaccine? An in-depth study of this issue would be of great relevance in guiding the next phase of the COVID-19 booster vaccination program.

The reasons for vaccine hesitancy are thought to be complex and case specific. The existing literature cites concern about COVID-19 vaccine safety and efficacy, vaccination accessibility, the severity of the regional epidemic, experience with routine vaccination, and guardians’ willingness to vaccinate their children as the primary reasons for hesitancy. For example, in a study of Canadians, individuals with vaccine hesitancy had a higher prevalence of being concerned about vaccine side effects [19]. The antecedents of vaccine hesitancy range from a lack of knowledge and awareness to culturally rooted reservations [20]. The most commonly reported reasons parents chose not to vaccinate their child against COVID-19 were concerns about long-term adverse side effects and a negative reaction [21]. At the same time, concern about side effects is also the most common reason for COVID-19 vaccine hesitancy in low- and middle-income countries [3]. In addition, misinformation and disinformation can substantially result in vaccine hesitancy [22] and might influence the vaccine advice of health care providers [23]. These factors will probably influence people’s hesitancy about the COVID-19 booster vaccination.

Understanding local barriers to vaccination and concerns about vaccination is critical to developing tailored interventions [24]. The current basic vaccination rate of COVID-19 vaccine in China exceeds 89% [12], which is probably one of the leading countries in the world not only because of its large population but also its extensive exchanges and closer cooperation with other regions [25,26]. However, the coverage of booster vaccination in China is not widespread, which is still a significant gap compared with the basic vaccination rate. Furthermore, compared to other countries around the world, the epidemic prevention and control policies in mainland China are adequate, and vaccines are supplied for free and in sufficient quantities [27], but the problem of vaccine hesitancy remains unresolved. Focusing on the problem of vaccine hesitancy in COVID-19 booster vaccination in mainland China will be highly representative. The existing research on booster vaccination hesitancy is limited, and hence, we designed a multicenter study focusing on COVID-19 vaccine booster vaccination and regular booster vaccination in mainland China. This study aimed to understand the current COVID-19 vaccine hesitancy in booster vaccination of the Chinese population and analyze the dynamic trend of vaccine hesitancy as well as its influencing factors.

## Methods

### Procedures and Participants

From June 29, 2022, to July 2, 2022, we used a self-designed questionnaire to perform a preliminary survey by recruiting web-based volunteers. We conducted The Dynamic Evolution of COVID-19 Vaccination Study, a national, multicenter, observational household tracking survey from China using a stratified random sampling method. Eastern, Central, Western, and Northeast geographical regions within mainland China were selected to form the sample for this study. Therefore, 4 cities were selected from the Eastern (Changzhou and Jiangsu), Central (Zhengzhou, Henan), Western (Xi’ning and Qinghai), and Northeast (Mudanjiang and Heilongjiang) regions.

We calculated the minimum sample size of each region according to the population proportion of China’s Seventh National Population Census. In each province, the sample size of urban and rural areas was determined according to the proportion of population in the Seventh National Population Census, more than 2 cities and 2 rural areas were randomly selected for sampling. For all of the cities and families in the sample, coding was carried out followed by random sampling. Finally, all members of the selected family have been involved in this survey (age≥18 years) and completed the web-based or offline questionnaire with the assistance of the investigators. A total of 2556 households from 4 geographic regions of China were enrolled in the survey.

The study was designed as a 4-stage survey. For the first stage, we conducted the survey from August 3, 2022, to August 14, 2022. Data collection is still ongoing for the second, third, and fourth stages. Here, we describe the results from the first stage of the study only.

### Ethics Approval

Participants were informed of the benefits and risks of participating in this study, and they provided informed consent. All data were used merely for research purposes. This study was approved by the Life Science Ethics Review Committee of Zhengzhou University (2021-01-12-05).

### Questionnaire Design and Data Collection

Web-based questionnaires were designed to collect data through a survey conducted through the Wenjuanxing platform. We also conducted a face-to-face interview with participants who cannot use a smartphone. The questionnaire covered five thematic areas: (1) sociodemographic characteristics: age, gender, nation, religion, marital status, educational status, subjective social status in China or community, smoking status, drinking status, and physical activity; (2) health conditions: chronic disease, the history of allergies, self-report health condition (measured by EuroQol 5 Dimensions Questionnaire [28-30]); (3) perception of COVID-19 and its vaccine: public health prevention measures, awareness of COVID-19 vaccines, the channel of accessing information, the way of accessing information, the risk of COVID-19 infection, the history of COVID-19 infection, the convenience of vaccination, and the uptake of COVID-19 vaccine; (4) self-perception (Kaiser-Meyer-Olkin=0.796): perceived severity (Cronbach α=.879), perceived susceptibility (Cronbach α=.865), perceived benefits (Cronbach α=.907), perceived barriers (Cronbach α=.927), and self-efficiency (Cronbach α=.913; Kaiser-Meyer-Olkin=0.886); and (5) trust in medical staff and developers. All questionnaires are shown in Multimedia Appendix 1.

### Assessments

**Figure 1:**
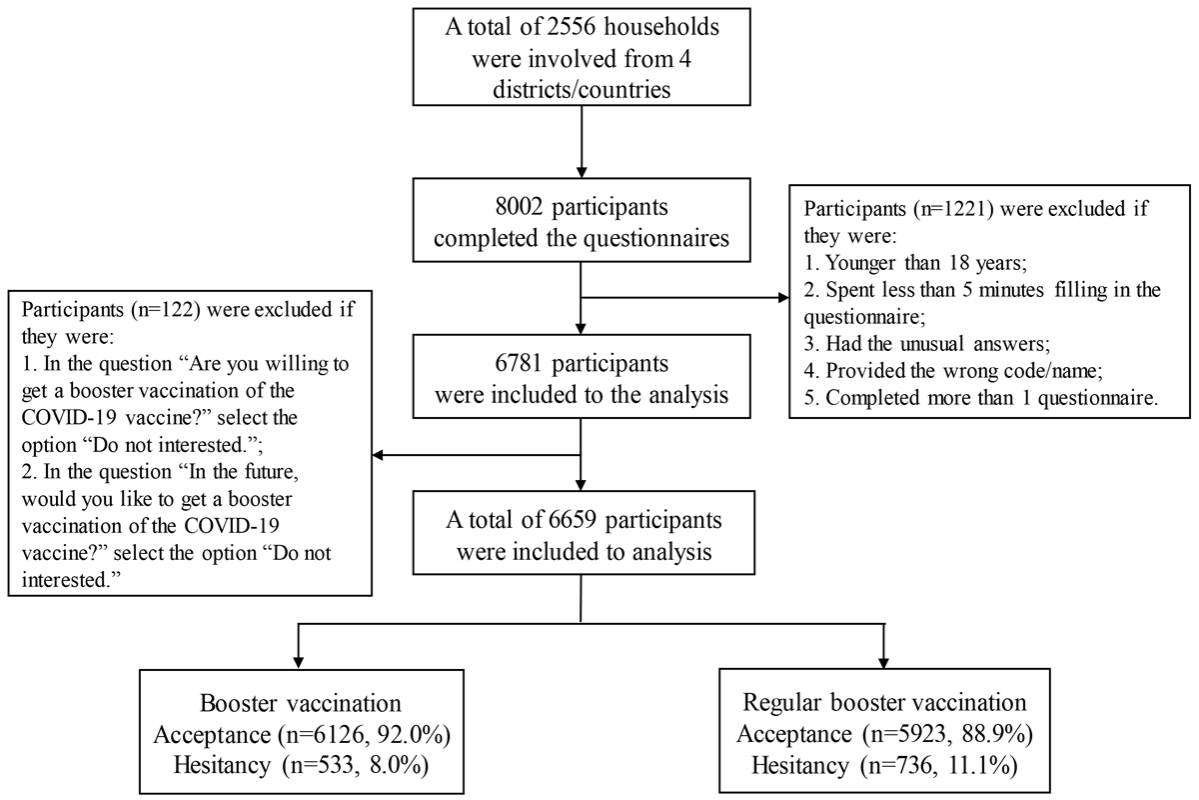
Flowchart of data processing and analysis.

Questionnaires with abnormal answers (contradictions and inconsistencies, eg, inconsistency or contradictions of sex) were excluded. In addition, we also excluded the participants who spent less than 5 minutes completing this questionnaire. Moreover, the remaining questionnaires were carefully reviewed by trained staff, and finally, 6659 questionnaires were deemed eligible for inclusion. The flowchart of participants is shown in Figure 1.

The primary outcome was COVID-19 vaccine hesitancy in both booster and regular booster vaccination, assessed by asking participants “Are you willing to get a booster vaccination of the COVID-19 vaccine?” and “In the future, would you like to get a booster vaccination of the COVID-19 vaccine?” Participants could choose 1 response from the options “(1) willing,” “(2) hesitant or delayed,” “(3) refused,” and “(4) do not interested” from the 2 questions. According to the definition of vaccine hesitancy, option 1 was regarded as “acceptance,” and options 2 and 3 were merged into “hesitancy.” In addition to this, participants were excluded if they selected the option “do not interested” (n=122).

A booster vaccination is a shot that is given after a period of time (which may be 6 months or more) after a population has completed a standard immunization program, and due to the gradual decay of antibodies in the body, the specific immunity in the body needs to be boosted with another booster vaccination. Regular booster vaccinations are administered after a period of booster vaccination to strengthen the immune system.

### Statistical Analysis

Chi-square tests were used to test differences in vaccine hesitancy between groups. Binary logistic regression analyses were carried out to examine factors associated with COVID-19 vaccine hesitancy in both booster and regular booster vaccination. A sensitivity analysis was performed by excluding the participants aged ≥80 years to test the robustness of results and assess the source of uncertainty. In stratified multistage sampling, post hoc stratification was used to weight the sample and the corresponding variables so that the sample's estimate of the total was unbiased. Odds ratios, 95% CI, and *P* values were calculated for each independent variable. All statistical analyses were carried out using SPSS (version 21.0; IBM Corp) and STATA (version 16.0; StataCorp). Differences were regarded as statistically significant if *P* values were less than .05.

## Results

### Characteristics of COVID-19 Vaccine Hesitancy in Booster Vaccination

Among the 8002 participants who completed the questionnaire, a total of 1343 participants who did not meet the inclusion criteria were subsequently excluded. Importantly, no statistically significant differences were found between the final 6659 participants included in the analysis and the overall sample of 8002 (*P*>.05) (Multimedia Appendix 2). The sociodemographic characteristics, awareness of COVID-19 vaccine, trust in the health care system as well as COVID-19 vaccine hesitancy in booster vaccination and regular booster vaccination of all study participants are displayed in Table 1. Overall, 533 adults (8%; 95% CI 7.4%-8.7%) showed a clear COVID-19 vaccine hesitancy in booster vaccination, while 6126 adults (92%; 95% CI 90.3%-94.2%) indicated that they were willing to vaccinate. Meanwhile, 736 participants (11.1%; 95% CI 10.3%-11.8%) expressed their hesitancy about regular booster vaccination of COVID-19 vaccine. Furthermore, we can see clearly that the participants of all ages are evenly distributed, the proportion of female participants (53.2%) is more than male (46.8%), and most of the participants (87.5%) were married.

**Figure 2:**
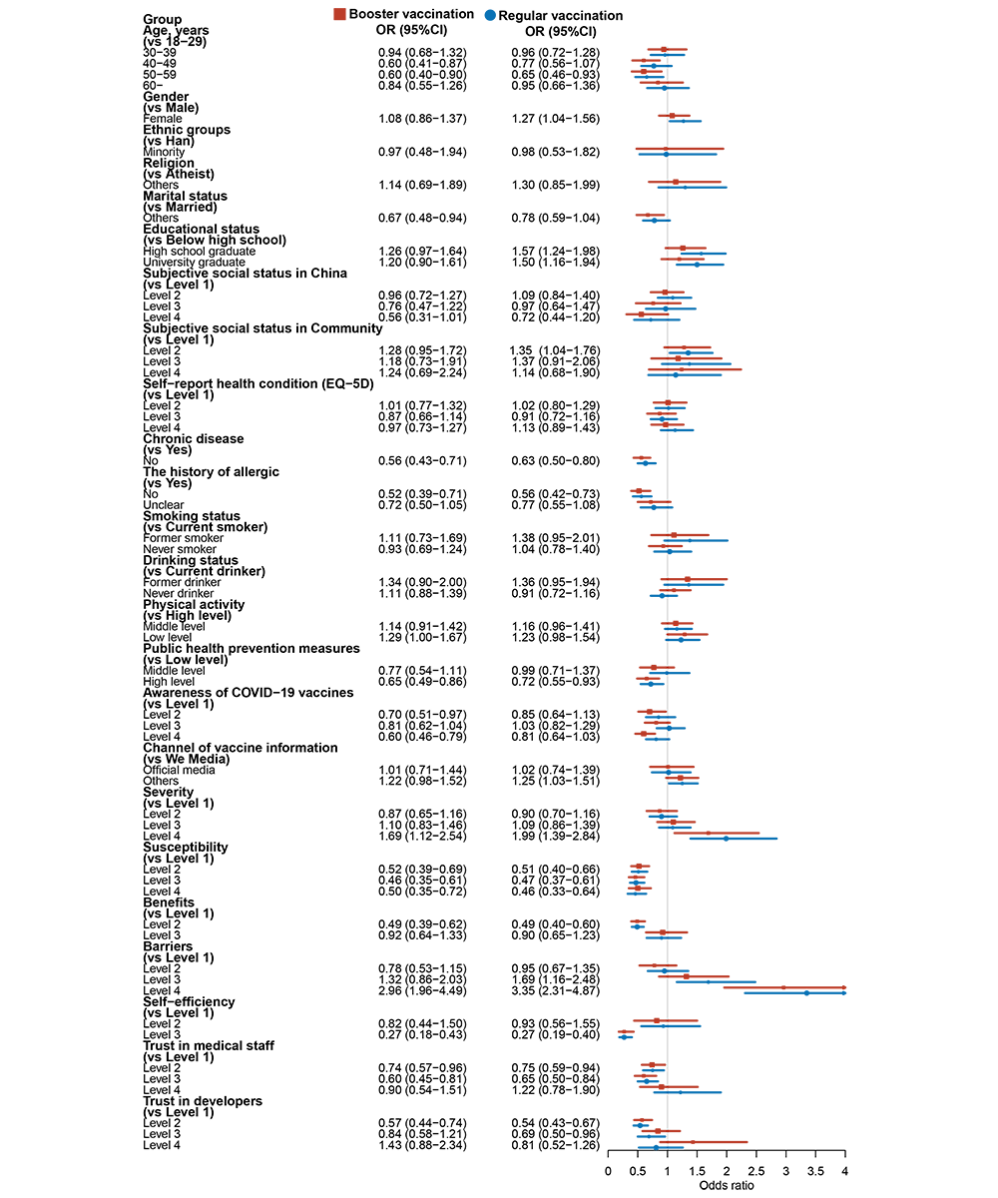
Influencing factors of vaccine hesitancy in both booster and regular booster vaccinations.

A higher prevalence of vaccine hesitancy in both booster vaccination and regular booster vaccination was observed among participants with a history of allergies, experiencing chronic disease, lower levels of public health prevention measures or susceptibility or benefits or self-efficiency, higher levels of severity or barriers, and lower trust in both medical staff and vaccine developers (all *P*<.05; Figure 2).

**Table 1:** The characteristics and COVID-19 vaccine booster hesitancy of all study participants in China (n=6659).

Covariates			Total, n (%)		*P* value^a^	Vaccine hesitancy in booster vaccination (95% CI)^b^		*P* value		Vaccine hesitancy in regular booster vaccination (95% CI)		*P* value
Total participants			6659 (100)			8.0 (7.4-8.7)				11.1 (10.3-11.8)		
**Age (years)**					<.001			<.001				<.001
	18-29	872 (13.1)				7.8 (6.0-9.6)			11.0 (8.9-13.1)			
	30-39	1801 (27)				9.6 (8.2-10.9)			12.8 (11.3-14.5)			
	40-49	1277 (19.2)				6.2 (4.9-7.7)			9.9 (8.2-11.5)			
	50-59	1408 (21.1)				6.0 (4.7-7.2)			8.2 (6.8-9.7)			
	≥60	1301 (19.5)				10.0 (8.4-11.6)			12.9 (11.1-14.7)			
**Gender**					<.001			.16				.02
	Male	3119 (46.8)				7.5 (6.6-8.5)			10.1 (9.0-11.2)			
	Female	3540 (53.2)				8.5 (7.5-9.4)			11.9 (10.9-13.0)			
**Ethnic groups**					<.001			.42				.27
	Han	6472 (97.2)				8.1 (7.4-8.7)			11.1 (10.4-11.9)			
	Minority	187 (2.8)				6.4 (2.9-9.9)			8.6 (4.6-12.6)			
**Religion**					<.001			.54				.88
	Atheist	6362 (95.5)				8.1 (7.4-8.7)			11.1 (10.3-11.8)			
	Others	297 (4.5)				7.1 (4.2-10.0)			10.8 (7.3-14.3)			
**Marital status**					<.001			.12				.43
	Married	5829 (87.5)				8.2 (7.5-8.9)			11.2 (10.4-12.0)			
	Others	830 (12.5)				6.6 (4.9-8.3)			10.2 (8.2-12.3)			
**Educational status**					<.001			<.001				<.001
	Below high school	2882 (43.3)				6.6 (5.7-7.5)			8.4 (7.4-9.4)			
	High school graduate	1661 (24.9)				8.1 (6.8-9.4)			11.9 (10.4-13.5)			
	University graduate	2116 (31.8)				9.8 (8.6-11.1)			14.0 (12.5-15.5)			
**Subjective social status in China**					<.001			.01				.003
	Level 1^c^	1942 (29.2)				7.8 (6.6-9.0)			9.9 (8.6-11.4)			
	Level 2^c^	2825 (42.4)				9.0 (7.9-10.0)			12.4 (11.2-13.6)			
	Level 3^c^	796 (12.0)				7.9 (6.0-9.8)			11.9 (9.7-14.2)			
	Level 4^c^	1096 (16.5)				5.8 (4.5-7.2)			8.9 (7.2-10.5)			
**Subjective social status in community**					<.001			.01				<.001
	Level 1^c^	1734 (26.0)				7.5 (6.3-8.8)			9.3 (7.9-10.7)			
	Level 2^c^	2896 (43.5)				9.2 (8.1-10.2)			12.7 (11.5-13.9)			
	Level 3^c^	858 (12.9)				7.6 (5.8-9.4)			11.9 (9.7-14.2)			
	Level 4^c^	1171 (17.6)				6.2 (4.9-7.6)			9.0 (7.3-10.6)			
**Self-report health condition (EQ-5D)^d^**					0.01			<.001				<.001
	Level 1^c^	1709 (25.7)				13.0 (11.4-14.7)			16.3 (14.5-18.1)			
	Level 2^c^	1627 (24.4)				7.9 (6.6-9.3)			11.0 (9.5-12.6)			
	Level 3^c^	1746 (26.2)				5.7 (4.6-6.9)			9.5 (8.1-10.9)			
	Level 4^c^	1577 (23.7)				5.3 (4.2-6.4)			7.2 (6.0-8.5)			
**Chronic disease**					<.001			<.001				<.001
	Yes	1030 (15.5)				6.9 (6.3-7.6)			17.1 (14.8-19.4)			
	No	5269 (84.5)				13.9 (11.8-16.0)			10.0 (9.2-10.7)			
**History of allergies**					<.001			<.001				<.001
	Yes	468 (7.0)				17.1 (13.7-20.5)			21.8 (18.1-25.5)			
	No	5466 (82.1)				6.6 (5.9-7.3)			9.4 (8.6-10.1)			
	Unclear	725 (10.9)				12.8 (10.4-15.3)			17.0 (14.2-19.7)			
**Smoking status**					<.001			.33				.05
	Current smoker	1432 (21.5)				8.0 (6.6-9.5)			9.8 (8.2-11.3)			
	Former smoker	402 (6.0)				10.0 (7.0-13.3)			13.9 (10.6-17.7)			
	Never smoker	4825 (72.5)				7.9 (7.1-8.6)			11.2 (10.3-12.1)			
**Drinking status**					<.001			.04				.03
	Current drinker	1706 (25.6)				7.8 (6.5-9.1)			10.7 (9.3-12.2)			
	Former drinker	387 (5.8)				11.4 (8.2-14.5)			15.3 (11.7-18.8)			
	Never drinker	4566 (68.6)				7.8 (7.0-8.6)			10.8 (9.9-11.7)			
**Physical activity**					<.001			<.001				<.001
	High level	3399 (51.0)				6.4 (5.6-7.2)			9.0 (8.0-9.9)			
	Middle level	2111 (31.7)				8.9 (7.7-10.1)			12.4 (11.0-13.8)			
	Low level	1149 (17.3)				11.1 (9.3-13.1)			14.8 (12.7-16.9)			
**Public health prevention measures**					<.001			<.001				<.001
	Low level	522 (7.8)				16.9 (13.7-20.1)			20.3 (16.9-23.8)			
	Middle level	527 (7.9)				14.2 (11.3-17.2)			20.9 (17.4-24.3)			
	High level	5610 (84.2)				6.6 (6.0-7.2)			9.3 (8.5-10.0)			
**Awareness of COVID-19 vaccines**					<.001			.03				.35
	Level 1^c^	2533 (38.3)				9.2 (8.1-10.4)			11.5 (10.2-12.7)			
	Level 2^c^	900 (13.5)				6.8 (5.1-8.4)			9.7 (7.7-11.6)			
	Level 3^c^	1593 (23.9)				8.0 (6.6-9.3)			11.7 (10.1-13.3)			
	Level 4^c^	1613 (24.2)				6.9 (5.7-8.1)			10.5 (9.0-12.0)			
**Channel of vaccine information**					<.001			.25				.15
	We Media	1818 (27.3)				8.9 (7.6-10.2)			12.3 (10.8-13.9)			
	Official media	704 (10.6)				7.7 (5.7-9.6)			10.2 (8.0-12.5)			
	Others	4137 (62.1)				7.7 (6.9-8.5)			10.7 (9.7-11.6)			
**Severity**					<.001			<.001				<.001
	Level 1^c^	1818 (27.3)				6.1 (5.0-7.2)			8.6 (7.3-9.9)			
	Level 2^c^	1556 (23.4)				10.3 (8.8-11.8)			14.3 (12.6-16.2)			
	Level 3^c^	2108 (31.7)				9.7 (8.5-11.1)			13.1 (11.7-14.6)			
	Level 4^c^	1177 (17.7)				4.8 (3.6-6.1)			6.8 (5.4-8.2)			
**Susceptibility**					<.001			.006				<.001
	Level 1^c^	1794 (26.9)				6.6 (5.5-7.8)			8.7 (7.4-10.0)			
	Level 2^c^	1889 (28.4)				8.1 (6.9-9.3)			11.3 (9.9-12.7)			
	Level 3^c^	2028 (30.5)				9.6 (8.3-10.9)			13.6 (12.1-15.1)			
	Level 4^c^	948 (14.2)				7.1 (5.4-8.7)			9.6 (7.7-11.5)			
**Benefits**					<.001			<.001				<.001
	Level 1^c^	1784 (26.8)				15.9 (14.2-17.6)			21.2 (19.3-23.1)			
	Level 2^c^	2765 (41.5)				6.6 (5.6-7.5)			9.6 (8.5-10.7)			
	Level 3^c^	2110 (31.7)				3.2 (2.5-4.0)			4.4 (3.5-5.3)			
**Barriers**					<.001			<.001				<.001
	Level 1^c^	2026 (30.4)				2.6 (1.9-3.3)			3.0 (2.3-3.8)			
	Level 2^c^	2453 (36.8)				4.4 (3.6-5.3)			6.8 (5.8-7.8)			
	Level 3^c^	740 (11.1)				10.8 (8.6-13.1)			16.6 (13.9-19.3)			
	Level 4^c^	1440 (21.6)				25.5 (23.3-27.6)			26.8 (24.5-29.1)			
**Self-efficiency**					<.001			<.001				<.001
	Level 1^c^	4328 (65.0)				11.4 (10.4-12.4)			15.7 (14.6-16.8)			
	Level 2^c^	191 (2.9)				6.8 (3.2-10.4)			10.5 (6.1-14.8)			
	Level 3^c^	2140 (32.1)				1.3 (0.8-1.7)			1.7 (1.2-2.3)			
**Trust in medical staff**					<.001			<.001				<.001
	Level 1^c^	2022 (30.4)				15.6 (14.0-17.2)			21.0 (19.2-22.7)			
	Level 2^c^	1314 (19.7)				7.8 (6.4-9.3)			10.9 (9.2-12.8)			
	Level 3^c^	1743 (26.2)				4.7 (3.7-5.7)			6.9 (5.7-8.1)			
	Level 4^c^	1580 (23.7)				2.1 (1.9-2.8)			3.1 (2.3-4.0)			
**Trust in developers**					<.001			<.001				<.001
	Level 1^c^	1783 (26.8)				17.2 (15.5-19.1)			23.6 (21.6-25.5)			
	Level 2^c^	2152 (32.3)				6.2 (5.2-7.3)			9.3 (8.1-10.5)			
	Level 3^c^	1194 (17.9)				4.4 (3.2-5.5)			6.0 (4.7-7.4)			
	Level 4^c^	1530 (23.0)				2.6 (1.8-3.4)			2.9 (2.0-3.7)			

^a^Differences between categories within each variable.

^b^Row percentages derived from the total number in the corresponding row.

^c^Levels 1-4 indicate progressively higher degrees. The higher the degree, the higher level of social status in China or community, the better the self-assessment of health status, the more awareness of COVID-19 vaccine, the more severe or more barriers, the greater the susceptibility or benefits, the higher the self-efficacy and the more trust in medical staff and developers.

^d^EQ-5D: EuroQol 5 dimensions.

### Factors Associated With COVID-19 Vaccine Hesitancy in Booster Vaccination

In the binary logistic regression model, subjective social status in China or community, chronic disease, history of allergies, drinking status, physical activity, public health prevention measures, awareness of COVID-19 vaccines, severity, susceptibility, benefits, barriers, self-efficiency, and trust in medical staff or developers were found to be all independently associated with COVID-19 vaccine hesitancy in both booster vaccination and regular booster vaccination (all *P*<.05).

After adjusting for potential confounders, we found that unmarried, experiencing chronic disease, history of allergies, low level of physical activity, lower level of public health prevention measures or susceptibility or benefits or self-efficiency, higher level of severity or barriers, and lower trust in medical staff or developers were all positively associated with COVID-19 vaccine hesitancy in booster vaccination (all *P*<.05). Meanwhile, we observed that participants who were female, with higher educational level, higher level of subjective social status in community, experiencing chronic disease, history of allergies, lower level of public health prevention measures or susceptibility or benefits or self-efficiency, higher level of severity or barriers, lower trust in both medical staff and developers were all positively associated with COVID-19 vaccine hesitancy in regular booster vaccination after adjusting for potential confounders (all *P*<.05). Detailed results are all shown in Figure 2 and Multimedia Appendix 3.

**Figure 3:**
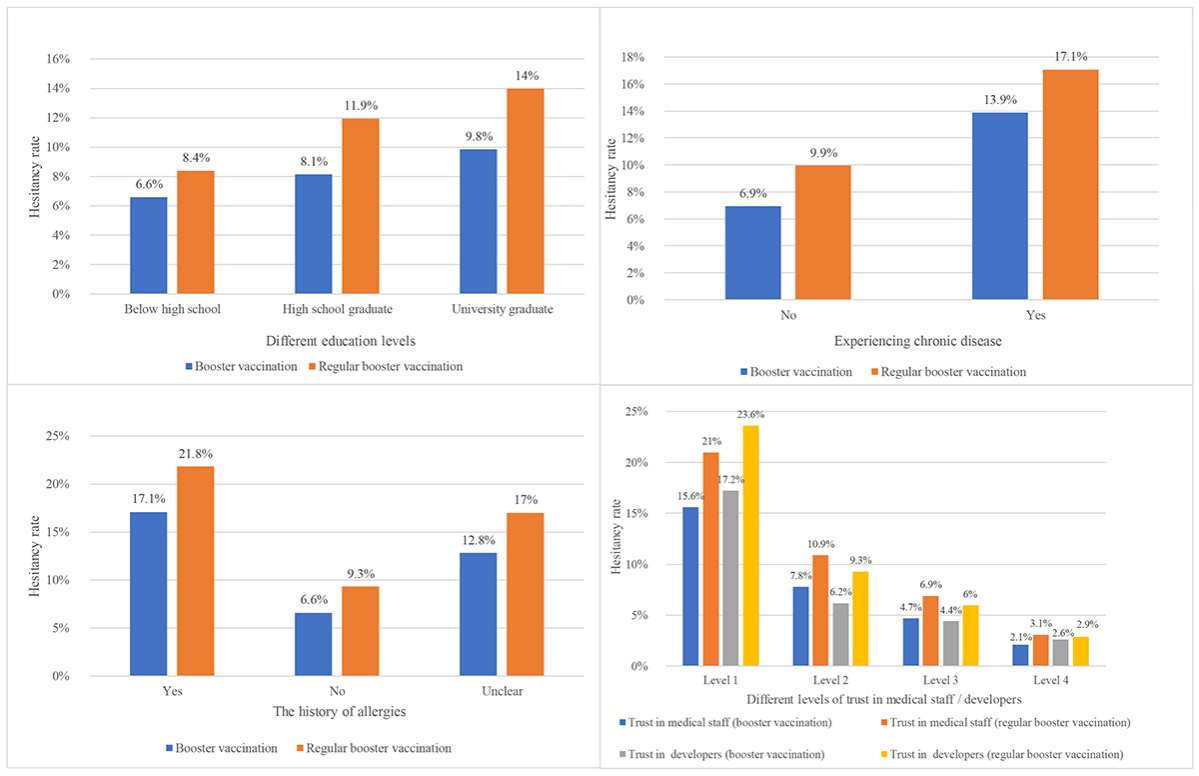
COVID-19 vaccine hesitancy rates for different influencing factors in both booster and regular booster vaccination.

In particular, participants with higher levels of education and lower levels of trust in medical staff or developers showed stronger COVID-19 vaccine hesitancy and higher rates of hesitancy at the following regular booster vaccination. The same trend could be observed in participants with a history of vaccination allergy and those with chronic disease (Figure 3).

### Dynamic Fluctuations of COVID-19 Vaccine Hesitancy in Booster Vaccination

The COVID-19 vaccine hesitancy rate among Chinese residents shows a trend of raising, which was from 8% (533/6659) to 11.1% (736/6659) between the booster vaccination and regular booster vaccination. In addition, some participants’ attitudes toward COVID-19 vaccine booster vaccination were shown to change. There were 5870 and 480 participants who remained vaccinated and hesitant about routine booster vaccination respectively, while there were still 256 participants who would switch from acceptance to hesitancy.

**Figure 4:**
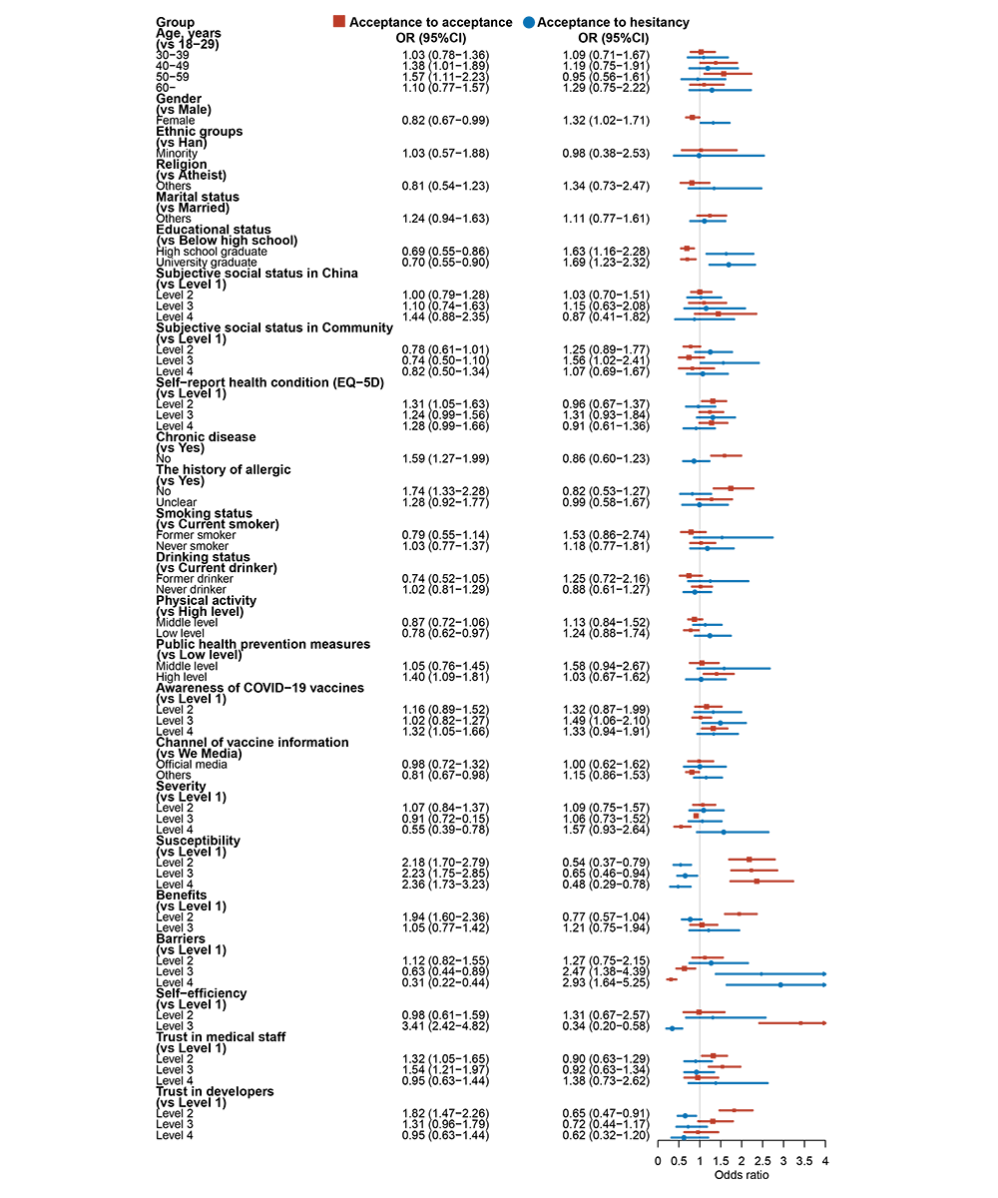
Relevant influences on acceptance to acceptance and acceptance to hesitancy for all participants.

**Figure 5:**
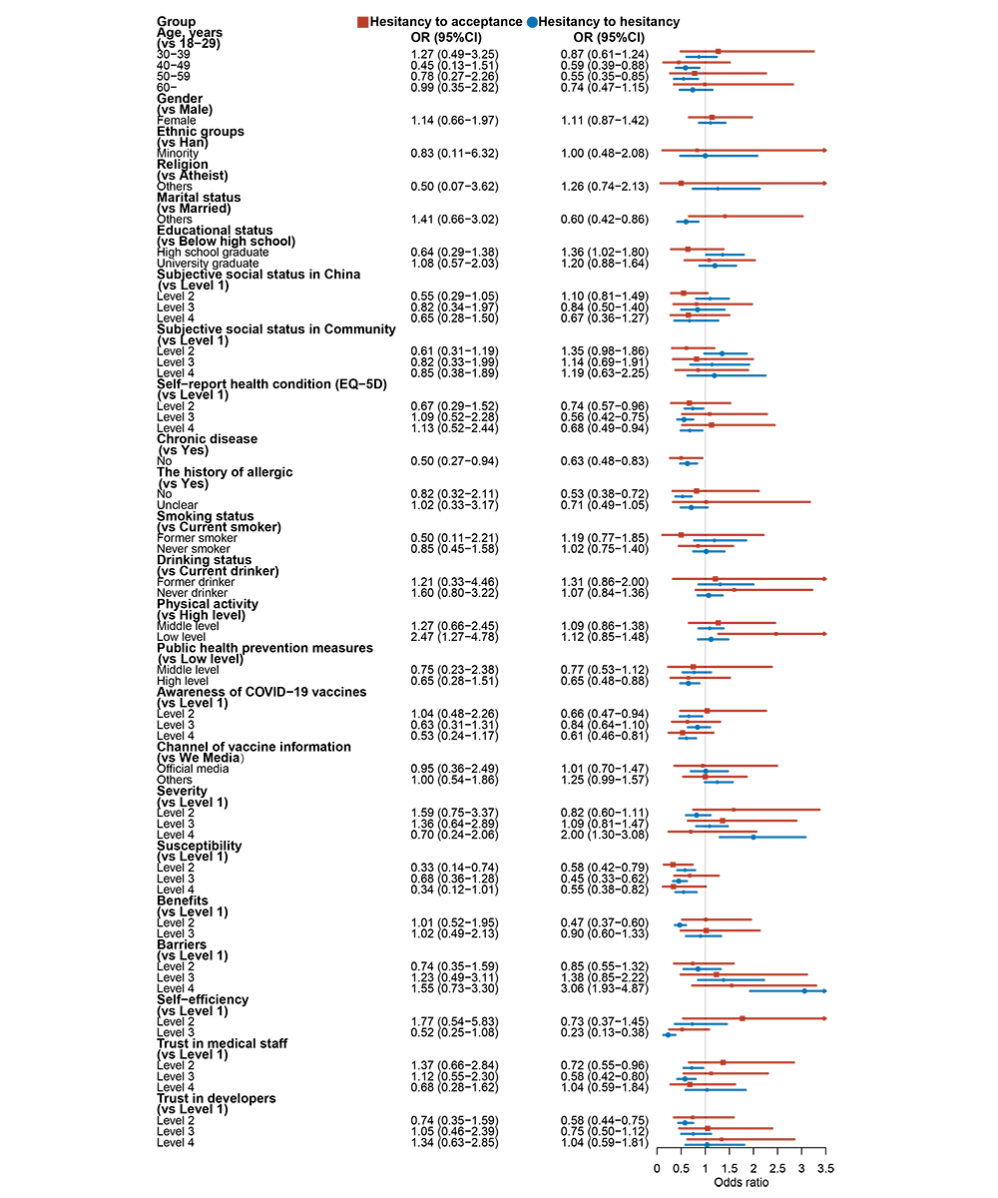
Relevant influences on hesitancy to acceptance and hesitancy to hesitancy for all participants.

Multimedia Appendix 4 lists 4 attitudinal changes toward COVID-19 booster vaccination, such as acceptance to acceptance, acceptance to hesitancy (ATH), hesitancy to acceptance, and hesitancy to hesitancy (HTH). The results demonstrated that participants who were female, with higher educational level, higher level of barriers, lower level of susceptibility or self-efficiency, and lower trust in developers were positively associated with ATH (all *P*<.05). Participants with higher levels of physical activity and self-efficiency as well as not experiencing chronic disease expressed their willingness from hesitancy to acceptance (all *P*<.001). Furthermore, participants who had higher educational level, lower self-report health condition, having from chronic disease, history of allergies, lower level of public health prevention measures or susceptibility or benefits or self-efficiency, higher level of barriers, lower trust in both medical staff and developers were all positively associated with HTH after adjusting for potential confounders (all *P*<.05). Detailed results are all presented in Figures 4 and 5.

### Sensitivity Analysis

In sensitivity analyses, the exclusion of participants aged >80 years did not significantly alter the outcome of COVID-19 vaccine hesitancy. Effect estimates for the primary outcome remained similar (Multimedia Appendix 5).

## Discussion

### Principal Findings

This study focused on the hesitancy rate of COVID-19 vaccine booster in the sample from 4 provinces in mainland China, namely Heilongjiang, Jiangsu, Henan, and Qinghai. The vast majority of participants (6126/6659, 92%) indicated a willingness to receive the COVID-19 booster vaccine, with an additional 8% (533/6659) expressing vaccine hesitancy. The higher vaccination rate and lower vaccine hesitancy rate may be attributed to the following reasons: first, China has continued to strengthen postmarketing surveillance of vaccines, emphasizing their safety and efficacy while continuously tracking the incidence of vaccine-preventable diseases and public acceptance of vaccines [26]. Second, the Chinese government has increased the supply of COVID-19 vaccines and set up reasonably designated medical institutions to carry out booster vaccinations, which has improved the accessibility of vaccination services [31]. Finally, the government and communities have used instant communication media such as television, news, and radio to promote the idea that the overall benefits of COVID-19 vaccination outweigh the risks, and disseminated scientific knowledge about vaccination to reduce people’s doubts. At the same time, community staff actively carried out door-to-door campaigns to mobilize residents actively participate in the booster vaccination.

Nevertheless, the hesitancy rate altered dynamically, increasing from 8% (533/6659) to 11% (736/6659) at the next regular COVID-19 booster vaccination. This may be related to the rapid mutation of the COVID-19 virus [10,32] and spot outbreaks in some cities such as Shanghai, Hainan, Zhengzhou, and so forth in mainland China [33]. People are beginning to wonder about the necessity of the COVID-19 booster vaccination and whether it is effective and safe. This result suggests that repeated outbreaks or spot outbreaks are destroying confidence in the COVID-19 vaccine, which will not be conducive to the control of the epidemic. Therefore, it would be relevant to explore the main influences on vaccine hesitancy and its dynamic fluctuations.

Previous studies have indicated that participants with a lower education level expressed a higher level of COVID-19 vaccine hesitancy in a global survey of 17 countries [34], adults in Ontario with less than a bachelor degree were more likely to report unwillingness to COVID-19 vaccination [35], as well as Chinese adults with lower education level endorsed clear vaccine hesitancy response in primary COVID-19 vaccination [26]. However, our findings suggest the opposite view. Those participants with higher levels of education were more reluctant to participate in the regular COVID-19 booster vaccination, showing a clear vaccine hesitancy. Previous studies have demonstrated similar results [36,37]. Likewise, a similar trend was observed in the regular and the booster vaccination attitude conversion (ATH and HTH). The reason for the contrary findings could be attributed to the fact that individuals with a higher level of education may have greater access to information regarding the COVID-19 outbreak and vaccine. This may lead them to believe that the epidemic will come to an end soon and that they will no longer require a booster shot for COVID-19 [5,20]. Alternatively, the highly educated population may have more resources to combat the risk of the COVID-19 epidemic [38,39].

It is worth noting that there was a significant association between experiencing chronic disease, history of previous vaccination allergies, and participants’ hesitancy to receive booster vaccinations. This hesitancy was also found in the conversion of attitudes from booster vaccination to regular booster vaccination. Participants with chronic diseases (hypertension, diabetes, etc) expressed clear hesitancy to receive booster vaccinations and were consistently hesitant to receive regular booster vaccinations. The reason for this consequence may be that people with chronic disease were concerned about whether people with immunodeficiency and immunosuppression should be vaccinated [40,41]. They are confused as to whether having a chronic disease can exacerbate adverse reactions to COVID-19 booster vaccination [42,43]. The same trend can be observed in participants with a history of vaccination allergy. Previous experiences with vaccination allergies have made people wary of the COVID-19 vaccine [44]. They are unsure if they will develop allergic injuries after the COVID-19 booster vaccination [45], so hesitancy about the booster is inevitable. Consequently, widespread education of people would be essential to reduce vaccine hesitancy.

In this study, we found that participants with higher levels of epidemic severity, barriers, and lower levels of susceptibility, benefits, and self-efficacy were more hesitant to receive a booster vaccination. The severity of the epidemic influenced people’s choice, with higher levels of severity instead promoting vaccine hesitancy, which is inconsistent with previous findings [12,46]. This may be due to people being more aware of self-protection, taking more adequate self-protection measures, going out less, or participating in timely nucleic acid testing when the epidemic is severe [47,48]. Although most people have confidence in the vaccine, this attitude does not automatically translate into vaccine use, as there are a variety of other barriers to vaccination [49,50]. While barriers to vaccination (eg, inconvenience and long queues) affect booster vaccination rates, a reasonable number of booster vaccination sites can facilitate vaccination [38], and the current high rate of COVID-19 vaccination in China can be attributed to a large number of vaccination sites. Therefore, in order to increase the booster vaccination rate, it is important to improve the accessibility of vaccination services. Besides, effective measures can be taken simultaneously, including ensuring the supply of vaccines, increasing the number of vaccination staff, improving the vaccination experience, and providing appointment services to reduce the waiting time for vaccination [26,50,51]. Consistent with the results of Włodarczyk and Ziętalewicz [52], higher self-efficacy and the benefits of COVID-19 vaccination influenced people to take part in vaccination, suggesting that we need to expand the perception that the benefits of vaccination outweigh the disadvantages and guide people positively to take part in vaccination with full self-efficacy.

Mistrust of medical staff and vaccine developers is an important factor influencing hesitancy in receiving COVID-19 booster vaccination. This has been demonstrated in previous studies of vaccine hesitancy [53-55]. Low levels of trust in medical staff and developers can lead to hesitancy in receiving COVID-19 booster vaccinations, even regular booster vaccinations. Both experiences of adverse reactions to vaccinations and even the influence of counterfeit vaccines in society have led to suspicion of medical staff and developers [56,57], which directly influences the COVID-19 booster vaccination. Therefore, it is imperative to strengthen the regulation of vaccine development, production, transportation, and vaccination. Strict adherence to industry guidelines and ethical standards will strongly ensure the standardization, safety, and efficacy of vaccines [16,58]. At the same time, it is necessary to strengthen the training of medical staff to enhance the communication between medical staff and residents, which aims to improve the quality of vaccination services. In addition, enhanced screening of patients with contraindications to COVID-19 vaccination is necessary [24,59], and avoiding or delaying vaccination of contraindicated patients will effectively reduce the occurrence and adverse effects of allergic events.

### Strengths and Limitations

This is the first multicenter nationwide household-based survey to assess the hesitancy of COVID-19 booster vaccination, influencing factors, and its dynamic fluctuation trend with the help of a saturation sample, which is highly innovative and forward-looking. Throughout the survey process, dedicated staff were assigned to follow-up the survey sites in each of the 4 centers, and strict quality control was carried out to ensure that the survey data were complete and authentic. However, there are still several limitations of this study. First, this study is a cross-sectional observational study, the causality association of vaccine hesitancy cannot be established, and it needs to be supported by data from the follow-up surveys. Second, it may introduce reporting bias in the collecting data on COVID-19 booster vaccination. Finally, this survey was conducted on a household-based sample with a population of permanent residents, and the findings may not be applicable to the floating population.

### Conclusions

The prevalence of COVID-19 vaccine booster hesitancy is not high in mainland China. However, there is a slight increment in hesitancy on regular booster vaccination. To reduce vaccine hesitancy, targeted information guidance for people with higher education levels and chronic diseases would be fruitful. Furthermore, improving the accessibility of booster vaccination and enhancing people’s trust in medical staff and vaccine developers would be highly effective in reducing vaccine hesitancy.

## Appendix

Multimedia Appendix 1 Original questionnaire: data collection and explanatory variables.

Multimedia Appendix 2 Analysis of variance between the participants (n=6659) included in the analysis and the total sample (N=8002).

Multimedia Appendix 3 The effect factors of vaccine hesitancy in both the booster and regular booster vaccination (n=6659).

Multimedia Appendix 4 Associations between COVID-19 booster vaccination acceptance, hesitancy transitions, and characteristics of all participants (n=6659).

Multimedia Appendix 5 The sensitivity analyses of factors influence booster and regular booster vaccination (n=6239).

## Abbreviations

ATH: acceptance to hesitancy
HTH: hesitancy to hesitancy
WHO: World Health Organization

## Data Availability

The data sets generated and analyzed during this study are available from the corresponding author on reasonable request.
