# Supplementary material for: Dynamic Trends and Underlying Factors of COVID-19 Vaccine Booster Hesitancy in Adults: Cross-Sectional Observational Study
Source: JMIR Public Health Surveill. 2023 Aug 1;9:e44822. doi: 10.2196/44822 (PMC10395646; doi:10.2196/44822)
Supplement: Multimedia Appendix 1 [file publichealth_v9i1e44822_app1.docx]

# Section 1. Personal information questionnaire

# I. General Information

| 1. Province City County (District) Township (Community) Village (Street) |
| --- |
| 1. The code __________ |
| 1. The name |
| 1. The telephone number _______. |
| 1. How old are you？ Age? |
| 1. What's your height in centimeters？ cm（centimeter） How many kilograms do you weigh？ kg（kilogram） |
| 1. What's your gender？ A. male B. female |
| 1. What is your religion？ 2. Not religious B. Buddhism C. Christian D. Taoism E. Islam F. Other |
| 1. What is your marital status?   A. Unmarried B. Married C. Divorced D. Death of a spouse E. Other |
| 1. What is your education level?   A. Illiteracy B. Primary and below C. Junior high school D. Senior High School (Technical secondary school) E. University and above |
| 1. How many people are there in your family？ People，including minors (＜18 years old) ______, elderly (≥65 years old) ______. |
| 1. 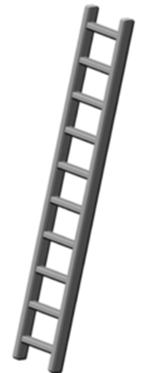At the top of the ladder are the richest -- they have the most money, the most education, the most respected jobs. At the bottom of the ladder are the worst off -- those with the least money, the least education, the least respected jobs or no jobs at all. The higher up the ladder you are, the closer you are to the people at the top; The lower you go, the closer you are to the people at the bottom. Please mark the steps with an "X" (0 at the bottom, 10 at the top) where you think you are (relative to other Chinese citizens). |
| 1. People define community in different ways; Please define it in whatever way makes most sense to you. At the top of the ladder are the highest ranking people in the community. At the bottom of the ladder are the lowest ranking people in the community. Ask you to mark the 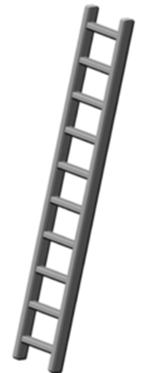ladder with an "X" (0 at the bottom, 10 at the top) where you think you are (relative to others in your community). |

# II. Lifestyle behavior

| 1. Do you maintain a reasonable weight？   A. Always B. Often C. Sometimes D. Little E. Never |
| --- |
| 1. Do you control unhealthy diet such as high salt and fat？   A. Always B. Often C. Sometimes D. Little E. Never |
| 1. Do you smoke (including e-cigarettes)？   A. Always B. Often C. Sometimes D. Little E. Have to give up smoking F. Never smoking |
| 1. Do you drink alcohol？   A. Always B. Often C. Sometimes D. Little E. Have to stop drinking F. Never drank |
| 1. How do you participate in light or moderate intensity physical activity? Such as walking, square dancing, etc)   A. Always B. Often C. Sometimes D. Little E. Never |
| 1. Do you pay attention to psychological decompression? Yoga, singing, holidays, etc)   A. Always B. Often C. Sometimes D. Little E. Never |
| 1. Are you constantly studying or working？   A. Always B. Often C. Sometimes D. Little E. Never |
| 1. Do you have regular health check-ups (excluding those for illness)？   A. Always B. Often C. Sometimes D. Little E. Never |

# III．Health condition and Quality of life Assessment (EQ-5D)

| 1. Do you currently have a chronic medical condition that has been diagnosed by a doctor or hospital？   A. Yes B. No |
| --- |
| 1. What is the main disease you have？   Hypertension □ Year of first diagnosis： year  Diabetes □ Year of first diagnosis： year  Other Diseases □ Year of first diagnosis： year |
| 1. Do you have a history of allergies？ A Yes B No |
| 1. Do you have any difficulty getting around today？   A. No difficulty B. A little bit difficult C. Moderate difficulty D. Be in serious difficulty E. Unable to get around |
| 1. Did you have trouble doing everyday activities like washing or dressing today？   A. No difficulty B. A little bit difficult C. Moderate difficulty D. Be in serious difficulty E. Unable to bathe or dress themselves |
| 1. Do you have any difficulty in your work, study, housework, leisure activities today？   A. No difficulty B. A little bit difficult C. Moderate difficulty D. Be in serious difficulty E. Inability to carry out daily activities |
| 1. Do you feel any pain or discomfort today？   A. No pain B. A little bit sore C. Moderate pain or discomfort D. Severe pain or discomfort E. Very severe pain or discomfort |
| 1. Are you feeling anxious or depressed today？   A. No anxiety or depression B. A little anxiety or depression C. Moderate anxiety or depression D. Have severe anxiety or depression E. Very severe anxiety or depression |
| 1. If 0 is the worst and 100 is the best, how would you rate your health today？   \|-----\|—---\|—---\|—---\|—---\|—---\|—---\|—---\|—---\|—---\|  0 10 20 30 40 50 60 70 80 90 100  Please write the number you marked on the scale in the space below.  Your health today = points |

**Section 2. Risk of infection exposure**

| 1. Nowadays, what is the COVID-19 risk level in your community/village?   A High risk B Medium Risk C Low Risk |
| --- |
| 1. How about wearing a mask, washing hands and social distancing?   A. No B. Occasionally C. Sometimes D. Often E. Always |

**Section 3. COVID-19 vaccine awareness and approaches**

| 1. Which of the following statements do you think is true? (Multiple options available)  A People who have been infected with novel coronavirus should still receive the coronavirus vaccine.  B COVID-19 vaccine is effective in preventing severe illness, hospitalization, and death caused by the current novel coronavirus.  C Always take protective measures (wearing masks, washing hands, social distancing, etc.) even after being vaccinated against COVID-19.  D Different types of COVID-19 vaccines are safe and effective for booster and basic injections.  E They may also infect novel coronavirus after completing the entire course of COVID-19 vaccination.  F In most people, the vaccine is effective against severe illness and death for at least six months.  G 4 to 6 months after the completion of the basic injection, you can receive a booster injection of COVID-19 vaccine to protect against severe disease.  H While taking antibiotics, you can get the COVID-19 vaccine.  I Women who are planning to be pregnant, pregnant or breastfeeding can be vaccinated against COVID-19. |
| --- |
| 1. Which of the following channels do you obtain information about the COVID-19 booster vaccine?   A Wechat, QQ, Weibo, forum/community, network broadcast platform, network video platform, etc.  B television, radio, newspapers and periodicals and their new media applications and accounts Medical treatment.  C commercial portal websites and commercial news clients.  D health care professionals.  E members of communities or village committees.  F family members, friends, colleagues and other surrounding people.  G others _____. |

**Section 4. Self-perceived state**

|  | Strongly disagree | disagree | normal | agree | Strongly agree |
| --- | --- | --- | --- | --- | --- |
| 1) Infection with COVID-19 is fatal |  |  |  |  |  |
| 2) There are many sequelae after COVID-19 is cured |  |  |  |  |  |
| 3) Novel coronavirus infection will greatly affect life |  |  |  |  |  |
| 4) I have a good chance to contact novel coronavirus |  |  |  |  |  |
| 5) I am at high risk of contracting COVID-19 |  |  |  |  |  |
| 6) Inoculation with booster needles of COVID-19 vaccine can significantly reduce the risk of novel coronavirus infection |  |  |  |  |  |
| 7) Vaccination strengthens the health benefits of those around you |  |  |  |  |  |
| 8) Getting a booster shot of the COVID-19 vaccine will help me get back to my normal life before the pandemic |  |  |  |  |  |
| 9) I don't trust the manufacturers of the booster shot |  |  |  |  |  |
| 10) I don't trust the vaccinator of the booster shot |  |  |  |  |  |
| 11) I am concerned about the safety of the booster shot |  |  |  |  |  |
| 12) I am concerned about the effectiveness of the COVID-19 booster shot |  |  |  |  |  |

**Section 5. Self-efficiency**

|  | Strongly disagree | disagree | normal | agree | Strongly agree |
| --- | --- | --- | --- | --- | --- |
| 1) Even if it takes time for work or study, I will get a booster shot. |  |  |  |  |  |
| 2) Even if no one around me gets it, I will get a booster shot. |  |  |  |  |  |
| 3) Even if I have a fear of needles, I will get it. |  |  |  |  |  |
| 4) Even if I still need to take protective measures after I get the vaccine, I will get it. |  |  |  |  |  |

**Section 6. The trust in medical staff and developers**

|  | Strongly disagree | disagree | normal | agree | Strongly agree |
| --- | --- | --- | --- | --- | --- |
| **medical staff** | | | | | |
| 1) I trust the medical staff. |  |  |  |  |  |
| 2) The medical staff didn't really care about me. |  |  |  |  |  |
| 3) The medical staff have my best interests at heart. |  |  |  |  |  |
| 4) The medical staff don't respect me. |  |  |  |  |  |
| 5) The medical staff wanted to do their best. |  |  |  |  |  |
| 6) The medical people don't know about my life. |  |  |  |  |  |
| 7) Medical staff often make mistakes. |  |  |  |  |  |
| 8) Medics choose this profession for the money. |  |  |  |  |  |
| 9) The medical staff will give me the vaccine even if it's bad for me. |  |  |  |  |  |
| **developers** | | | | | |
| 1) Vaccine developers put vaccine safety first. |  |  |  |  |  |
| 2) I don't trust the vaccine developers. |  |  |  |  |  |
| 3) Vaccine developers just want to make money. |  |  |  |  |  |
| 4) Vaccine developers are not interested in helping others. |  |  |  |  |  |
| 5) Vaccine developers failed to properly check vaccine safety. |  |  |  |  |  |

**Section 7. Accessibility of COVID-19 booster vaccination services**

| 1. How far are you from the nearest booster vaccination site?  A less than 0.5km B 0.5-1km C 1.1-3km D 3.1-5km E greater than 5km F not clear |
| --- |
| 2. How long does it take you to get to the nearest booster vaccination site?  A less than 15 minutes B 15-30 minutes C 31-60 minutes D more than 1 hour F Unclear |

**Section 8. Vaccination against COVID-19**

| 1. Are you willing to receive a booster vaccination of the COVID-19 vaccine?  A willing B hesitant or delayed C refusing D do not interested |
| --- |
| 2. In the future, would you like to get a booster vaccination of COVID-19 vaccine regularly?  A willing B hesitant or delayed C refusing D do not interested |
| 3. What is the primary factor that affects your vaccination with the booster shot of COVID-19 vaccine?  A Suggestions from healthcare workers  B publicity from TV and newspapers  C publicity from new media  D publicity from celebrities  E policy incentives  F calls from the government, communities or work and study units  G persuasion from people around them  H the effect of the vaccine after vaccination  I experience of people around you infected with COVID-19  J the pressure of a large social population  K it doesn't matter |
| 4. Have you been vaccinated against COVID-19?  A Yes, I have received and completed fully vaccination  B Yes, I have received but not completed fully vaccination (skip to question 6)  C No, I have not received at all (skip to Section.11) |
| 5. When did you complete the basic vaccination of the COVID-19 vaccine? |
| 6. When did you receive the first dose of COVID-19 vaccine? |
| 7. Have you completed the booster vaccination of COVID-19 vaccine?  A Yes B no (skip to question 9) |
| 8. When did you finish the booster shot of COVID-19 vaccine? |
| 9. What is the main reason why you have not received a booster vaccination? (Skip to Section 11)  A Not meeting the conditions for vaccination (such as personal health reasons, not reaching the vaccination interval, etc.)  B Concerns about vaccine safety  C Concerns about vaccine effectiveness  D Distance from the inoculation site  E is confident of fighting COVID-19 infection  F believes the outbreak will end soon  G There is no vaccine at the vaccination site  H Low risk of infection  I The protection cycle of the booster needle is short  J Fear of needles  K Information on booster shot is not well understood  L Other reasons _____ |

**Section 9. Satisfaction with COVID-19 booster vaccination service**

| items | Strongly disagree | disagree | normal | agree | Strongly agree |
| --- | --- | --- | --- | --- | --- |
| 1) Ease of inoculation process |  |  |  |  |  |
| 2) Attitude towards vaccination service |  |  |  |  |  |
| 3) Waiting time for vaccination |  |  |  |  |  |
| 4) The environment around the inoculation site |  |  |  |  |  |
| 5) Inoculation transportation convenience degree |  |  |  |  |  |
| 6) The whole process of vaccination |  |  |  |  |  |

**Section 10. Effect of COVID-19 vaccine booster vaccination**

| 1. Have you had any serious adverse reactions after receiving the booster shot of COVID-19 vaccine? A yes B no |
| --- |
| 2. Have you been infected with novel coronavirus after receiving the booster vaccine? (including asymptomatic infection and confirmed diagnosis) A yes B no |

**Section 11. Guardians' intention to vaccinate adolescents (3-17 years old) with the COVID-19 booster vaccination**

| 1. How many teenagers (3-17 years old) are there in your family?   A None (end) B 1 C 2 D 3 or more |
| --- |
| 1. Are you a teenager?   A father B mother C grandfather D grandmother E grandfather F grandmother G other |
| 1. Do you think that being infected with COVID-19 will cause serious damage to his or her health?   A perfectly possible B possible C uncertain, D unlikely E completely impossible |
| 1. Do you think he/she is at high risk of contracting COVID-19?   A Very high risk B high risk C medium risk D low risk E no risk |
| 1. Do you think that getting a booster vaccination of COVID-19 vaccine can reduce his or her risk of contracting COVID-19?   A perfectly possible B possible C uncertain, D unlikely E completely impossible |
| 1. Are you concerned about the safety of his or her vaccine booster shot?   A very worried B worried C general D unworried E completely unworried |
| 1. Are you concerned about the effectiveness of the COVID-19 booster shot?   A very worried B worried C general D unworried E completely unworried |
| 1. Are you worried about the adverse reactions of him/her receiving the booster vaccination of COVID-19 vaccine?   A very worried B worried C general D unworried E completely unworried |
| 1. Do you think it is important to strengthen his or her health with the COVID-19 vaccine?   A very important B important C general D unimportant E not important at all |
| 1. Has he/she been vaccinated against COVID-19?   A Yes, and the basic vaccination of COVID-19 vaccine has been completed  B Yes, but the basic vaccination of COVID-19 vaccine has not been completed  C no |
| 1. Are you willing to let him/her get a booster shot of COVID-19 vaccine?   A willing B uncertain but prefers to vaccinate C uncertain but prefers not to vaccinate (jump to question 12) D unwilling (jump to question 12) |
| 1. What is the main reason why you are not willing/inclined to let him/her get a booster shot of COVID-19 vaccine?   A concern about vaccine safety  B concern about vaccine effectiveness  C too young age  D low risk of infection  E insufficient information on booster vaccination for adolescents  F short protection cycle  G I do not believe that novel coronavirus will lead to severe diseases  H outbreaks can be effectively controlled I Tend to have him or her take other measures to prevent infection (such as wearing a mask, Social distancing, etc.)  J Check whether teenagers in the surrounding families have been vaccinated  K specific anti-Covid-19 drugs have been marketed  L physical reasons of teenagers (such as history of vaccine allergy)  M vaccination is too troublesome (such as distance from the vaccination point, etc.)  N He (she)/they have fear of needles  O others |
