# Supplementary material for: Dynamic Trends and Underlying Factors of COVID-19 Vaccine Booster Hesitancy in Adults: Cross-Sectional Observational Study
Source: JMIR Public Health Surveill. 2023 Aug 1;9:e44822. doi: 10.2196/44822 (PMC10395646; doi:10.2196/44822)
Supplement: Multimedia Appendix 2 [file publichealth_v9i1e44822_app2.docx]

| **Covariates** | **8002 participants** | **6659 participants** | ***P* value** |
| --- | --- | --- | --- |
| Age, years | | | |
| 18-29 | 1093(13.7) | 872(13.1) | 0.833 |
| 30-39 | 2178(27.2) | 1801(27.0) |  |
| 40-49 | 1521(19.0) | 1277(19.2) |  |
| 50-59 | 1688(21.1) | 1408(21.1) |  |
| ≥60 | 1522(19.0) | 1301(19.5) |  |
| Gender | | | |
| Male | 3791(47.4) | 3119(46.8) | 0.517 |
| Female | 4211(52.6) | 3540(53.2) |  |
| Ethnic groups | | | |
| Han | 7782(97.3) | 6472(97.2) | 0.841 |
| Minority | 220(2.7) | 187(2.8) |  |
| Religion | | | |
| Atheist | 7648(95.6) | 6362(95.5) | 0.936 |
| Others | 354(4.4) | 297(4.5) |  |
| Educational status | | | |
| Below high school | 3438(43.0) | 2882(43.3) | 0.541 |
| High school graduate | 2059(25.7) | 1661(24.9) |  |
| University graduate | 2505(31.3) | 2116(31.8) |  |
