# Supplementary material for: Dynamic Trends and Underlying Factors of COVID-19 Vaccine Booster Hesitancy in Adults: Cross-Sectional Observational Study
Source: JMIR Public Health Surveill. 2023 Aug 1;9:e44822. doi: 10.2196/44822 (PMC10395646; doi:10.2196/44822)
Supplement: Multimedia Appendix 3 [file publichealth_v9i1e44822_app3.docx]

| **Covariates** | **Vaccine hesitancy in booster vaccination** | | | | **Vaccine hesitancy in regular booster vaccination** | | | |
| --- | --- | --- | --- | --- | --- | --- | --- | --- |
|  | **OR(95%CI)^a^** | ***P-*value** | **OR(95%CI)^b^** | ***P-*value** | **OR(95%CI)^a^** | ***P-*value** | **OR(95%CI)^c^** | ***P-*value** |
| Age, years |  |  |  |  |  |  |  |  |
| 18-29 | 1.00(ref.) |  | 1.00(ref.) |  | 1.00(ref.) |  | 1.00(ref.) |  |
| 30-39 | 1.09(0.80-1.48) | 0.580 | 0.94(0.68-1.32) | 0.734 | 1.11(0.85-1.44) | 0.451 | 0.96(0.72-1.28) | 0.788 |
| 40-49 | 0.63(0.44-0.89) | 0.008 | 0.60(0.41-0.87) | 0.008 | 0.76(0.57-1.01) | 0.058 | 0.77(0.56-1.07) | 0.120 |
| 50-59 | 0.68(0.49-0.96) | 0.026 | 0.60(0.40-0.90) | 0.014 | 0.67(0.50-0.89) | 0.006 | 0.65(0.46-0.93) | 0.018 |
| 60- | 1.05(0.77-1.44) | 0.756 | 0.84(0.55-1.26) | 0.394 | 0.99(0.76-1.31) | 0.992 | 0.95(0.66-1.36) | 0.782 |
| Gender |  |  |  |  |  |  |  |  |
| Male | 1.00(ref.) |  | 1.00(ref.) |  | 1.00(ref.) |  | 1.00(ref.) |  |
| Female | 1.09(0.91-1.30) | 0.368 | 1.08(0.86-1.37) | 0.491 | 1.17(1.00-1.38) | 0.045 | 1.27(1.04-1.56) | 0.021 |
| Ethnic groups |  |  |  |  |  |  |  |  |
| Han | 1.00(ref.) |  | 1.00(ref.) |  | 1.00(ref.) |  | 1.00(ref.) |  |
| Minority | 1.48(0.79-2.78) | 0.223 | 0.97(0.48-1.94) | 0.933 | 1.50(0.86-2.61) | 0.150 | 0.98(0.53-1.82) | 0.958 |
| Religion |  |  |  |  |  |  |  |  |
| Atheist | 1.00(ref.) |  | 1.00(ref.) |  | 1.00(ref.) |  | 1.00(ref.) |  |
| Others | 1.23(0.77-1.96) | 0.383 | 1.14(0.69-1.89) | 0.610 | 1.36(0.92-2.02) | 0.119 | 1.30(0.85-1.99) | 0.229 |
| Marital status |  |  |  |  |  |  |  |  |
| Married | 1.00(ref.) |  | 1.00(ref.) |  | 1.00(ref.) |  | 1.00(ref.) |  |
| Others | 0.79(0.59-1.06) | 0.113 | 0.67(0.48-0.94) | 0.019 | 0.90(0.71-1.15) | 0.411 | 0.78(0.59-1.04) | 0.085 |
| Educational status |  |  |  |  |  |  |  |  |
| Below high school | 1.00(ref.) |  | 1.00(ref.) |  | 1.00(ref.) |  | 1.00(ref.) |  |
| High school graduate | 1.17(0.92-1.47) | 0.199 | 1.26(0.97-1.64) | 0.086 | 1.39(1.13-1.70) | 0.001 | 1.57(1.24-1.98) | <0.001 |
| University graduate | 1.14(0.93-1.41) | 0.217 | 1.20(0.90-1.61) | 0.208 | 1.35(1.12-1.63) | 0.001 | 1.50(1.16-1.94) | 0.002 |
| Subjective social status in China |  |  |  |  |  |  |  |  |
| Level 1 | 1.00(ref.) |  | 1.00(ref.) |  | 1.00(ref.) |  | 1.00(ref.) |  |
| Level 2 | 0.93(0.75-1.15) | 0.503 | 0.96(0.72-1.27) | 0.763 | 1.04(0.86-1.26) | 0.686 | 1.09(0.84-1.40) | 0.513 |
| Level 3 | 0.81(0.59-1.12) | 0.202 | 0.76(0.47-1.22) | 0.256 | 0.99(0.76-1.29) | 0.935 | 0.97(0.64-1.47) | 0.900 |
| Level 4 | 0.55(0.40-0.75) | <0.001 | 0.56(0.31-1.01) | 0.053 | 0.66(0.51-0.86) | 0.002 | 0.72(0.44-1.20) | 0.211 |
| Subjective social status in Community | | | | | | | | |
| Level 1 | 1.00(ref.) |  | 1.00(ref.) |  | 1.00(ref.) |  | 1.00(ref.) |  |
| Level 2 | 1.01(0.81-1.27) | 0.902 | 1.28(0.95-1.72) | 0.111 | 1.17(0.96-1.44) | 0.119 | 1.35(1.04-1.76) | 0.026 |
| Level 3 | 0.84(0.61-1.15) | 0.276 | 1.18(0.73-1.91) | 0.488 | 1.09(0.83-1.43) | 0.518 | 1.37(0.91-2.06) | 0.135 |
| Level 4 | 0.63(0.47-0.86) | 0.003 | 1.24(0.69-2.24) | 0.476 | 0.75(0.57-0.97) | 0.030 | 1.14(0.68-1.90) | 0.612 |
| Self-report health condition (EQ-5D) | | | | | | | | |
| Level 1 | 1.00(ref.) |  | 1.00(ref.) |  | 1.00(ref.) |  | 1.00(ref.) |  |
| Level 2 | 0.97(0.75-1.24) | 0.791 | 1.01(0.77-1.32) | 0.943 | 0.98(0.78-1.22) | 0.831 | 1.02(0.80-1.29) | 0.870 |
| Level 3 | 0.90(0.70-1.16) | 0.428 | 0.87(0.66-1.14) | 0.304 | 0.93(0.75-1.16) | 0.532 | 0.91(0.72-1.16) | 0.454 |
| Level 4 | 0.98(0.76-1.26) | 0.858 | 0.97(0.73-1.27) | 0.809 | 1.11(0.89-1.38) | 0.348 | 1.13(0.89-1.43) | 0.333 |
| Chronic disease |  |  |  |  |  |  |  |  |
| Yes | 1.00(ref.) |  | 1.00(ref.) |  | 1.00(ref.) |  | 1.00(ref.) |  |
| No | 0.56(0.45-0.69) | <0.001 | 0.56(0.43-0.71) | <0.001 | 0.65(0.54-0.79) | <0.001 | 0.63(0.50-0.80) | <0.001 |
| The history of allergic |  |  |  |  |  |  |  |  |
| Yes | 1.00(ref.) |  | 1.00(ref.) |  | 1.00(ref.) |  | 1.00(ref.) |  |
| No | 0.42(0.32-0.55) | <0.001 | 0.52(0.39-0.71) | <0.001 | 0.44(0.35-0.56) | <0.001 | 0.56(0.42-0.73) | <0.001 |
| Unclear | 0.81(0.58-1.12) | 0.202 | 0.72(0.50-1.05) | 0.085 | 0.82(0.61-1.11) | 0.206 | 0.77(0.55-1.08) | 0.126 |
| Smoking status |  |  |  |  |  |  |  |  |
| Current smoker | 1.00(ref.) |  | 1.00(ref.) |  | 1.00(ref.) |  | 1.00(ref.) |  |
| Former smoker | 1.22(0.83-1.79) | 0.322 | 1.11(0.73-1.69) | 0.626 | 1.41(1.01-1.98) | 0.046 | 1.38(0.95-2.01) | 0.090 |
| Never smoker | 0.97(0.78-1.21) | 0.787 | 0.93(0.69-1.24) | 0.608 | 1.14(0.93-1.39) | 0.206 | 1.04(0.78-1.40) | 0.770 |
| Drinking status |  |  |  |  |  |  |  |  |
| Current drinker | 1.00(ref.) |  | 1.00(ref.) |  | 1.00(ref.) |  | 1.00(ref.) |  |
| Former drinker | 1.45(1.00-2.09) | 0.049 | 1.34(0.90-2.00) | 0.153 | 1.42(1.03-1.96) | 0.035 | 1.36(0.95-1.94) | 0.093 |
| Never drinker | 0.96(0.78-1.19) | 0.727 | 1.11(0.88-1.39) | 0.382 | 0.96(0.80-1.16) | 0.682 | 0.91(0.72-1.16) | 0.460 |
| Physical activity |  |  |  |  |  |  |  |  |
| High level | 1.00(ref.) |  | 1.00(ref.) |  | 1.00(ref.) |  | 1.00(ref.) |  |
| Middle level | 1.43(1.16-1.76) | 0.001 | 1.14(0.91-1.42) | 0.263 | 1.42(1.19-1.70) | <0.001 | 1.16(0.96-1.41) | 0.132 |
| Low level | 1.84(1.45-2.32) | <0.001 | 1.29(1.00-1.67) | 0.049 | 1.76(1.43-2.16) | <0.001 | 1.23(0.98-1.54) | 0.075 |
| Public health prevention measures | | | | | | | | |
| Low level | 1.00(ref.) |  | 1.00(ref.) |  | 1.00(ref.) |  | 1.00(ref.) |  |
| Middle level | 0.79(0.56-1.11) | 0.171 | 0.77(0.54-1.11) | 0.164 | 0.99(0.74-1.35) | 0.988 | 0.99(0.71-1.37) | 0.932 |
| High level | 0.42(0.32-0.54) | <0.001 | 0.65(0.49-0.86) | 0.003 | 0.49(0.38-0.62) | <0.001 | 0.72(0.55-0.93) | 0.014 |
| Awareness of COVID-19 vaccines | | | | | | | | |
| Level 1 | 1.00(ref.) |  | 1.00(ref.) |  | 1.00(ref.) |  | 1.00(ref.) |  |
| Level 2 | 0.62(0.46-0.83) | 0.002 | 0.70(0.51-0.97) | 0.030 | 0.73(0.56-0.94) | 0.016 | 0.85(0.64-1.13) | 0.256 |
| Level 3 | 0.60(0.47-0.76) | <0.001 | 0.81(0.62-1.04) | 0.099 | 0.74(0.61-0.91) | 0.004 | 1.03(0.82-1.29) | 0.783 |
| Level 4 | 0.47(0.36-0.60) | <0.001 | 0.60(0.46-0.79) | <0.001 | 0.61(0.49-0.75) | <0.001 | 0.81(0.64-1.03) | 0.086 |
| Channel of vaccine information |  |  |  |  |  |  |  |  |
| We Media | 1.00(ref.) |  | 1.00(ref.) |  | 1.00(ref.) |  | 1.00(ref.) |  |
| Official media | 0.82(0.59-1.14) | 0.238 | 1.01(0.71-1.44) | 0.961 | 0.80(0.60-1.06) | 0.123 | 1.02(0.74-1.39) | 0.910 |
| Others | 1.01(0.82-1.23) | 0.933 | 1.22(0.98-1.52) | 0.074 | 0.99(0.84-1.19) | 0.968 | 1.25(1.03-1.51) | 0.026 |
| Severity |  |  |  |  |  |  |  |  |
| Level 1 | 1.00(ref.) |  | 1.00(ref.) |  | 1.00(ref.) |  | 1.00(ref.) |  |
| Level 2 | 1.72(1.33-2.22) | <0.001 | 0.87(0.65-1.16) | 0.349 | 1.77(1.42-2.21) | <0.001 | 0.90(0.70-1.16) | 0.416 |
| Level 3 | 1.73(1.35-2.21) | <0.001 | 1.10(0.83-1.46) | 0.493 | 1.71(1.39-2.12) | <0.001 | 1.09(0.86-1.39) | 0.487 |
| Level 4 | 0.80(0.57-1.13) | 0.205 | 1.69(1.12-2.54) | 0.011 | 0.84(0.63-1.12) | 0.236 | 1.99(1.39-2.84) | <0.001 |
| Susceptibility |  |  |  |  |  |  |  |  |
| Level 1 | 1.00(ref.) |  | 1.00(ref.) |  | 1.00(ref.) |  | 1.00(ref.) |  |
| Level 2 | 1.16(0.90-1.50) | 0.247 | 0.52(0.39-0.69) | <0.001 | 1.24(0.99-1.54) | 0.063 | 0.51(0.40-0.66) | <0.001 |
| Level 3 | 1.42(1.12-1.82) | 0.004 | 0.46(0.35-0.61) | <0.001 | 1.58(1.28-1.96) | <0.001 | 0.47(0.37-0.61) | <0.001 |
| Level 4 | 1.06(0.77-1.45) | 0.732 | 0.50(0.35-0.72) | <0.001 | 1.11(0.84-1.47) | 0.457 | 0.46(0.33-0.64) | <0.001 |
| Benefits |  |  |  |  |  |  |  |  |
| Level 1 | 1.00(ref.) |  | 1.00(ref.) |  | 1.00(ref.) |  | 1.00(ref.) |  |
| Level 2 | 0.35(0.28-0.43) | <0.001 | 0.49(0.39-0.62) | <0.001 | 0.37(0.31-0.44) | <0.001 | 0.49(0.40-0.60) | <0.001 |
| Level 3 | 0.15(0.11-0.20) | <0.001 | 0.92(0.64-1.33) | 0.668 | 0.15(0.12-0.19) | <0.001 | 0.90(0.65-1.23) | 0.505 |
| Barriers |  |  |  |  |  |  |  |  |
| Level 1 | 1.00(ref.) |  | 1.00(ref.) |  | 1.00(ref.) |  | 1.00(ref.) |  |
| Level 2 | 1.95(1.39-2.74) | <0.001 | 0.78(0.53-1.15) | 0.214 | 2.56(1.89-3.46) | <0.001 | 0.95(0.67-1.35) | 0.787 |
| Level 3 | 4.11(2.85-5.93) | <0.001 | 1.32(0.86-2.03) | 0.207 | 5.77(4.17-7.99) | <0.001 | 1.69(1.16-2.48) | 0.007 |
| Level 4 | 10.75(7.88-14.65) | <0.001 | 2.96(1.96-4.49) | <0.001 | 13.13(9.87-17.48) | <0.001 | 3.35(2.31-4.87) | <0.001 |
| Self-efficiency |  |  |  |  |  |  |  |  |
| Level 1 | 1.00(ref.) |  | 1.00(ref.) |  | 1.00(ref.) |  | 1.00(ref.) |  |
| Level 2 | 0.52(0.29-0.92) | 0.026 | 0.82(0.44-1.50) | 0.517 | 0.58(0.36-0.94) | 0.028 | 0.93(0.56-1.55) | 0.786 |
| Level 3 | 0.09(0.06-0.13) | <0.001 | 0.27(0.18-0.43) | <0.001 | 0.09(0.06-0.12) | <0.001 | 0.27(0.19-0.40) | <0.001 |
| Trust in medical staff |  |  |  |  |  |  |  |  |
| Level 1 | 1.00(ref.) |  | 1.00(ref.) |  | 1.00(ref.) |  | 1.00(ref.) |  |
| Level 2 | 0.49(0.39-0.62) | <0.001 | 0.74(0.57-0.96) | 0.023 | 0.48(0.39-0.59) | <0.001 | 0.75(0.59-0.94) | 0.014 |
| Level 3 | 0.27(0.21-0.35) | <0.001 | 0.60(0.45-0.81) | 0.001 | 0.28(0.22-0.34) | <0.001 | 0.65(0.50-0.84) | 0.001 |
| Level 4 | 0.11(0.08-0.16) | <0.001 | 0.90(0.54-1.51) | 0.696 | 0.11(0.08-0.16) | <0.001 | 1.22(0.78-1.90) | 0.376 |
| Trust in developers |  |  |  |  |  |  |  |  |
| Level 1 | 1.00(ref.) |  | 1.00(ref.) |  | 1.00(ref.) |  | 1.00(ref.) |  |
| Level 2 | 0.32(0.26-0.40) | <0.001 | 0.57(0.44-0.74) | <0.001 | 0.33(0.28-0.40) | <0.001 | 0.54(0.43-0.67) | <0.001 |
| Level 3 | 0.23(0.17-0.31) | <0.001 | 0.84(0.58-1.21) | 0.347 | 0.21(0.16-0.28) | <0.001 | 0.69(0.50-0.96) | 0.026 |
| Level 4 | 0.12(0.08-0.17) | <0.001 | 1.43(0.88-2.34) | 0.150 | 0.09(0.06-0.12) | <0.001 | 0.81(0.52-1.26) | 0.343 |

OR, odds ratio; CI, confidence interval.

Levels 1-4: indicate progressively higher degrees. The higher the degree, the higher level of social status in China/community, the better the self-assessment of health status, the more awareness of COVID-19 vaccine, the more severe/barriers, the greater the susceptibility/benefits, the higher the self-efficacy and the more trust in medical staff and developers.

^a^ unadjusted;

^b^ adjusted age, educational status, subjective social status in China/community, self-report health condition (EQ-5D), chronic disease, the history of allergic, drinking status, physical activity, public health prevention measures, awareness of COVID-19 vaccines, severity, susceptibility, benefits, barriers, self-efficiency, the trust in medical staff, and the trust in developers.

^c^ adjusted age, gender, educational status, subjective social status in China/community, self-report health condition (EQ-5D), chronic disease, the history of allergic, drinking status, physical activity, public health prevention measures, severity, susceptibility, benefits, barriers, self-efficiency, the trust in medical staff, and the trust in developers.
