# Supplementary material for: Dynamic Trends and Underlying Factors of COVID-19 Vaccine Booster Hesitancy in Adults: Cross-Sectional Observational Study
Source: JMIR Public Health Surveill. 2023 Aug 1;9:e44822. doi: 10.2196/44822 (PMC10395646; doi:10.2196/44822)
Supplement: Multimedia Appendix 4 [file publichealth_v9i1e44822_app4.docx]

| **Covariates** | **Acceptance to acceptance(n=5870)** | | **Acceptance to hesitancy(n=256)** | | **Hesitancy to acceptance(n=53)** | | **Hesitancy to hesitancy(n=480)** | |
| --- | --- | --- | --- | --- | --- | --- | --- | --- |
|  | **OR(95%CI)^a^** | **OR(95%CI)^b^** | **OR(95%CI)^a^** | **OR(95%CI)^c^** | **OR(95%CI)^a^** | **OR(95%CI)^d^** | **OR(95%CI)^a^** | **OR(95%CI)^e^** |
| Age, years |  |  |  |  |  |  |  |  |
| 18-29 | 1.00(ref.) | 1.00(ref.) | 1.00(ref.) | 1.00(ref.) | 1.00(ref.) | 1.00(ref.) | 1.00(ref.) | 1.00(ref.) |
| 30-39 | 0.89(0.69-1.14) | 1.03(0.78-1.36) | 1.16(0.76-1.76) | 1.09(0.71-1.67) | 1.37(0.54-3.49) | 1.27(0.49-3.25) | 1.06(0.77-1.46) | 0.87(0.61-1.24) |
| 40-49 | 1.36(1.02-1.80) | 1.38(1.01-1.89) | 1.02(0.65-1.59) | 1.19(0.75-1.91) | 0.49(0.15-1.63) | 0.45(0.13-1.51) | 0.64(0.45-0.92) | 0.59(0.39-0.88) |
| 50-59 | 1.48(1.11-1.96) | 1.57(1.11-2.23) | 0.71(0.45-1.14) | 0.95(0.56-1.61) | 0.90(0.32-2.55) | 0.78(0.27-2.26) | 0.66(0.47-0.95) | 0.55(0.35-0.85) |
| 60- | 0.98(0.75-1.28) | 1.10(0.77-1.57) | 0.95(0.60-1.48) | 1.29(0.75-2.22) | 1.33(0.50-3.54) | 0.99(0.35-2.82) | 1.02(0.74-1.42) | 0.74(0.47-1.15) |
| Gender |  |  |  |  |  |  |  |  |
| Male | 1.00(ref.) | 1.00(ref.) | 1.00(ref.) | 1.00(ref.) | 1.00(ref.) | 1.00(ref.) | 1.00(ref.) | 1.00(ref.) |
| Female | 0.85(0.73-0.99) | 0.82(0.67-0.99) | 1.31(1.02-1.70) | 1.32(1.02-1.71) | 1.10(0.64-1.90) | 1.14(0.66-1.97) | 1.08(0.90-1.31) | 1.11(0.87-1.42) |
| Ethnic groups |  |  |  |  |  |  |  |  |
| Han | 1.00(ref.) | 1.00(ref.) | 1.00(ref.) | 1.00(ref.) | 1.00(ref.) | 1.00(ref.) | 1.00(ref.) | 1.00(ref.) |
| Minority | 0.69(0.40-1.18) | 1.03(0.57-1.88) | 1.25(0.49-3.16) | 0.98(0.38-2.53) | 0.95(0.13-7.17) | 0.83(0.11-6.32) | 1.55(0.80-2.98) | 1.00(0.48-2.08) |
| Religion |  |  |  |  |  |  |  |  |
| Atheist | 1.00(ref.) | 1.00(ref.) | 1.00(ref.) | 1.00(ref.) | 1.00(ref.) | 1.00(ref.) | 1.00(ref.) | 1.00(ref.) |
| Others | 0.77(0.52-1.13) | 0.81(0.54-1.23) | 1.33(0.73-2.43) | 1.34(0.73-2.47) | 0.54(0.07-3.91) | 0.50(0.07-3.62) | 1.32(0.82-2.13) | 1.26(0.74-2.13) |
| Marital status |  |  |  |  |  |  |  |  |
| Married | 1.00(ref.) | 1.00(ref.) | 1.00(ref.) | 1.00(ref.) | 1.00(ref.) | 1.00(ref.) | 1.00(ref.) | 1.00(ref.) |
| Others | 1.08(0.85-1.36) | 1.24(0.94-1.63) | 1.24(0.87-1.77) | 1.11(0.77-1.61) | 1.29(0.60-2.74) | 1.41(0.66-3.02) | 0.74(0.54-1.01) | 0.60(0.42-0.86) |
| Educational status |  |  |  |  |  |  |  |  |
| Below high school | 1.00(ref.) | 1.00(ref.) | 1.00(ref.) | 1.00(ref.) | 1.00(ref.) | 1.00(ref.) | 1.00(ref.) | 1.00(ref.) |
| High school graduate | 0.77(0.63-0.93) | 0.69(0.55-0.86) | 1.55(1.11-2.16) | 1.63(1.16-2.28) | 0.57(0.27-1.23) | 0.64(0.29-1.38) | 1.26(0.99-1.61) | 1.36(1.02-1.80) |
| University graduate | 0.76(0.64-0.91) | 0.70(0.55-0.90) | 1.63(1.20-2.21) | 1.69(1.23-2.32) | 0.88(0.48-1.61) | 1.08(0.57-2.03) | 1.18(0.95-1.47) | 1.20(0.88-1.64) |
| Subjective social status in China |  |  |  |  |  |  |  |  |
| Level 1 | 1.00(ref.) | 1.00(ref.) | 1.00(ref.) | 1.00(ref.) | 1.00(ref.) | 1.00(ref.) | 1.00(ref.) | 1.00(ref.) |
| Level 2 | 1.02(0.85-1.23) | 1.00(0.79-1.28) | 1.09(0.79-1.50) | 1.03(0.70-1.51) | 0.50(0.26-0.97) | 0.55(0.29-1.05) | 1.00(0.80-1.26) | 1.10(0.81-1.49) |
| Level 3 | 1.04(0.80-1.35) | 1.10(0.74-1.63) | 1.30(0.86-1.97) | 1.15(0.63-2.08) | 0.75(0.31-1.80) | 0.82(0.34-1.97) | 0.83(0.60-1.16) | 0.84(0.50-1.40) |
| Level 4 | 1.54(1.20-1.98) | 1.44(0.88-2.35) | 0.95(0.63-1.43) | 0.87(0.41-1.82) | 0.59(0.26-1.36) | 0.65(0.28-1.50) | 0.55(0.40-0.77) | 0.67(0.36-1.27) |
| Subjective social status in Community | | | | | | | | |
| Level 1 | 1.00(ref.) | 1.00(ref.) | 1.00(ref.) | 1.00(ref.) | 1.00(ref.) | 1.00(ref.) | 1.00(ref.) | 1.00(ref.) |
| Level 2 | 0.90(0.74-1.10) | 0.78(0.61-1.01) | 1.29(0.92-1.82) | 1.25(0.89-1.77) | 0.56(0.29-1.08) | 0.61(0.31-1.19) | 1.09(0.86-1.39) | 1.35(0.98-1.86) |
| Level 3 | 0.95(0.73-1.23) | 0.74(0.50-1.10) | 1.58(1.04-2.41) | 1.56(1.02-2.41) | 0.75(0.31-1.82) | 0.82(0.33-1.99) | 0.86(0.61-1.20) | 1.14(0.69-1.91) |
| Level 4 | 1.36(1.05-1.75) | 0.82(0.50-1.34) | 1.04(0.68-1.60) | 1.07(0.69-1.67) | 0.75(0.34-1.64) | 0.85(0.38-1.89) | 0.63(0.45-0.87) | 1.19(0.63-2.25) |
| Self-report health condition (EQ-5D) | | | | | | | | |
| Level 1 | 1.00(ref.) | 1.00(ref.) | 1.00(ref.) | 1.00(ref.) | 1.00(ref.) | 1.00(ref.) | 1.00(ref.) | 1.00(ref.) |
| Level 2 | 1.64(1.34-2.01) | 1.31(1.05-1.63) | 0.85(0.60-1.21) | 0.96(0.67-1.37) | 0.56(0.25-1.26) | 0.67(0.29-1.52) | 0.56(0.44-0.72) | 0.74(0.57-0.96) |
| Level 3 | 1.74(1.42-2.13) | 1.24(0.99-1.56) | 1.10(0.79-1.52) | 1.31(0.93-1.84) | 0.86(0.42-1.75) | 1.09(0.52-2.28) | 0.40(0.31-0.53) | 0.56(0.42-0.75) |
| Level 4 | 2.01(1.60-2.51) | 1.28(0.99-1.66) | 0.71(0.48-1.04) | 0.91(0.61-1.36) | 0.90(0.43-1.87) | 1.13(0.52-2.44) | 0.42(0.32-0.56) | 0.68(0.49-0.94) |
| Chronic disease |  |  |  |  |  |  |  |  |
| Yes | 1.00(ref.) | 1.00(ref.) | 1.00(ref.) | 1.00(ref.) | 1.00(ref.) | 1.00(ref.) | 1.00(ref.) | 1.00(ref.) |
| No | 1.59(1.32-1.91) | 1.59(1.27-1.99) | 0.92(0.66-1.27) | 0.86(0.60-1.23) | 0.51(0.27-0.96) | 0.50(0.27-0.94) | 0.57(0.46-0.72) | 0.63(0.48-0.83) |
| The history of allergic |  |  |  |  |  |  |  |  |
| Yes | 1.00(ref.) | 1.00(ref.) | 1.00(ref.) | 1.00(ref.) | 1.00(ref.) | 1.00(ref.) | 1.00(ref.) | 1.00(ref.) |
| No | 2.22(1.75-2.81) | 1.74(1.33-2.28) | 0.66(0.43-1.00) | 0.82(0.53-1.27) | 0.76(0.30-1.95) | 0.82(0.32-2.11) | 0.40(0.31-0.53) | 0.53(0.38-0.72) |
| Unclear | 1.20(0.90-1.61) | 1.28(0.92-1.77) | 0.98(0.59-1.63) | 0.99(0.58-1.67) | 1.06(0.34-3.27) | 1.02(0.33-3.17) | 0.79(0.56-1.12) | 0.71(0.49-1.05) |
| Smoking status |  |  |  |  |  |  |  |  |
| Current smoker | 1.00(ref.) | 1.00(ref.) | 1.00(ref.) | 1.00(ref.) | 1.00(ref.) | 1.00(ref.) | 1.00(ref.) | 1.00(ref.) |
| Former smoker | 0.76(0.54-1.05) | 0.79(0.55-1.14) | 1.51(0.85-2.67) | 1.53(0.86-2.74) | 0.50(0.11-2.21) | 0.50(0.11-2.21) | 1.32(0.89-1.97) | 1.19(0.77-1.85) |
| Never smoker | 0.91(0.75-1.10) | 1.03(0.77-1.37) | 1.43(1.01-2.02) | 1.18(0.77-1.81) | 0.78(0.42-1.44) | 0.85(0.45-1.58) | 1.00(0.79-1.27) | 1.02(0.75-1.40) |
| Drinking status |  |  |  |  |  |  |  |  |
| Current drinker | 1.00(ref.) | 1.00(ref.) | 1.00(ref.) | 1.00(ref.) | 1.00(ref.) | 1.00(ref.) | 1.00(ref.) | 1.00(ref.) |
| Former drinker | 0.71(0.51-0.97) | 0.74(0.52-1.05) | 1.25(0.73-2.15) | 1.25(0.72-2.16) | 1.29(0.35-4.73) | 1.21(0.33-4.46) | 1.45(0.99-2.12) | 1.31(0.86-2.00) |
| Never drinker | 1.01(0.84-1.20) | 1.02(0.81-1.29) | 1.06(0.78-1.43) | 0.88(0.61-1.27) | 1.50(0.75-3.02) | 1.60(0.80-3.22) | 0.92(0.74-1.14) | 1.07(0.84-1.36) |
| Physical activity |  |  |  |  |  |  |  |  |
| High level | 1.00(ref.) | 1.00(ref.) | 1.00(ref.) | 1.00(ref.) | 1.00(ref.) | 1.00(ref.) | 1.00(ref.) | 1.00(ref.) |
| Middle level | 0.71(0.59-0.84) | 0.87(0.72-1.06) | 1.29(0.97-1.71) | 1.13(0.84-1.52) | 1.22(0.63-2.34) | 1.27(0.66-2.45) | 1.45(1.17-1.80) | 1.09(0.86-1.38) |
| Low level | 0.55(0.45-0.67) | 0.78(0.62-0.97) | 1.56(1.12-2.16) | 1.24(0.88-1.74) | 2.28(1.18-4.39) | 2.47(1.27-4.78) | 1.76(1.37-2.25) | 1.12(0.85-1.48) |
| Public health prevention measures |  |  |  |  |  |  |  |  |
| Low level | 1.00(ref.) | 1.00(ref.) | 1.00(ref.) | 1.00(ref.) | 1.00(ref.) | 1.00(ref.) | 1.00(ref.) | 1.00(ref.) |
| Middle level | 1.03(0.76-1.38) | 1.05(0.76-1.45) | 1.58(0.94-2.65) | 1.58(0.94-2.67) | 0.71(0.22-2.24) | 0.75(0.23-2.38) | 0.80(0.56-1.14) | 0.77(0.53-1.12) |
| High level | 2.07(1.64-2.62) | 1.40(1.09-1.81) | 0.82(0.53-1.27) | 1.03(0.67-1.62) | 0.54(0.24-1.24) | 0.65(0.28-1.51) | 0.42(0.32-0.54) | 0.65(0.48-0.88) |
| Awareness of COVID-19 vaccines |  |  |  |  |  |  |  |  |
| Level 1 | 1.00(ref.) | 1.00(ref.) | 1.00(ref.) | 1.00(ref.) | 1.00(ref.) | 1.00(ref.) | 1.00(ref.) | 1.00(ref.) |
| Level 2 | 1.34(1.04-1.72) | 1.16(0.89-1.52) | 1.15(0.77-1.72) | 1.32(0.87-1.99) | 1.03(0.47-2.23) | 1.04(0.48-2.26) | 0.58(0.42-0.80) | 0.66(0.47-0.94) |
| Level 3 | 1.37(1.12-1.67) | 1.02(0.82-1.27) | 1.17(0.84-1.63) | 1.49(1.06-2.10) | 0.64(0.31-1.33) | 0.63(0.31-1.31) | 0.60(0.47-0.77) | 0.84(0.64-1.10) |
| Level 4 | 1.68(1.37-2.07) | 1.32(1.05-1.66) | 1.06(0.75-1.49) | 1.33(0.94-1.91) | 0.52(0.24-1.15) | 0.53(0.24-1.17) | 0.47(0.36-0.60) | 0.61(0.46-0.81) |
| Channel of vaccine information |  |  |  |  |  |  |  |  |
| We Media | 1.00(ref.) | 1.00(ref.) | 1.00(ref.) | 1.00(ref.) | 1.00(ref.) | 1.00(ref.) | 1.00(ref.) | 1.00(ref.) |
| Official media | 1.24(0.94-1.63) | 0.98(0.72-1.32) | 0.83(0.52-1.32) | 1.00(0.62-1.62) | 1.00(0.38-2.60) | 0.95(0.36-2.49) | 0.81(0.57-1.14) | 1.01(0.70-1.47) |
| Others | 1.00(0.85-1.19) | 0.81(0.67-0.98) | 0.98(0.74-1.30) | 1.15(0.86-1.53) | 1.02(0.55-1.89) | 1.00(0.54-1.86) | 1.01(0.82-1.24) | 1.25(0.99-1.57) |
| Severity |  |  |  |  |  |  |  |  |
| Level 1 | 1.00(ref.) | 1.00(ref.) | 1.00(ref.) | 1.00(ref.) | 1.00(ref.) | 1.00(ref.) | 1.00(ref.) | 1.00(ref.) |
| Level 2 | 0.56(0.46-0.70) | 1.07(0.84-1.37) | 1.67(1.18-2.36) | 1.09(0.75-1.57) | 1.50(0.72-3.10) | 1.59(0.75-3.37) | 1.73(1.32-2.27) | 0.82(0.60-1.11) |
| Level 3 | 0.59(0.48-0.73) | 0.91(0.72-0.15) | 1.45(1.03-2.03) | 1.06(0.73-1.52) | 1.22(0.59-2.51) | 1.36(0.64-2.89) | 1.78(1.38-2.31) | 1.09(0.81-1.47) |
| Level 4 | 1.23(0.93-1.63) | 0.55(0.39-0.78) | 0.88(0.55-1.40) | 1.57(0.93-2.64) | 0.57(0.20-1.65) | 0.70(0.24-2.06) | 0.84(0.59-1.20) | 2.00(1.30-3.08) |
| Susceptibility |  |  |  |  |  |  |  |  |
| Level 1 | 1.00(ref.) | 1.00(ref.) | 1.00(ref.) | 1.00(ref.) | 1.00(ref.) | 1.00(ref.) | 1.00(ref.) | 1.00(ref.) |
| Level 2 | 0.90(0.72-1.11) | 2.18(1.70-2.79) | 1.00(0.70-1.43) | 0.54(0.37-0.79) | 0.34(0.15-0.78) | 0.33(0.14-0.74) | 1.36(1.03-1.78) | 0.58(0.42-0.79) |
| Level 3 | 0.67(0.55-0.83) | 2.23(1.75-2.85) | 1.46(1.05-2.03) | 0.65(0.46-0.94) | 0.76(0.41-1.42) | 0.68(0.36-1.28) | 1.57(1.21-2.05) | 0.45(0.33-0.62) |
| Level 4 | 0.99(0.76-1.30) | 2.36(1.73-3.23) | 0.91(0.57-1.44) | 0.48(0.29-0.78) | 0.36(0.12-1.04) | 0.34(0.12-1.01) | 1.23(0.88-1.71) | 0.55(0.38-0.82) |
| Benefits |  |  |  |  |  |  |  |  |
| Level 1 | 1.00(ref.) | 1.00(ref.) | 1.00(ref.) | 1.00(ref.) | 1.00(ref.) | 1.00(ref.) | 1.00(ref.) | 1.00(ref.) |
| Level 2 | 2.62(2.21-3.11) | 1.94(1.60-2.36) | 0.59(0.45-0.78) | 0.77(0.57-1.04) | 0.90(0.46-1.73) | 1.01(0.52-1.95) | 0.32(0.26-0.40) | 0.47(0.37-0.60) |
| Level 3 | 5.94(4.72-7.48) | 1.05(0.77-1.42) | 0.31(0.21-0.44) | 1.21(0.75-1.94) | 0.84(0.42-1.70) | 1.02(0.49-2.13) | 0.12(0.09-0.17) | 0.90(0.60-1.33) |
| Barriers |  |  |  |  |  |  |  |  |
| Level 1 | 1.00(ref.) | 1.00(ref.) | 1.00(ref.) | 1.00(ref.) | 1.00(ref.) | 1.00(ref.) | 1.00(ref.) | 1.00(ref.) |
| Level 2 | 0.46(0.35-0.61) | 1.12(0.82-1.55) | 2.39(1.50-3.80) | 1.27(0.75-2.15) | 0.72(0.34-1.52) | 0.74(0.35-1.59) | 2.49(1.69-3.69) | 0.85(0.55-1.32) |
| Level 3 | 0.20(0.15-0.28) | 0.63(0.44-0.89) | 5.17(3.16-8.46) | 2.47(1.38-4.39) | 1.18(0.48-2.91) | 1.23(0.49-3.11) | 5.34(3.53-8.09) | 1.38(0.85-2.22) |
| Level 4 | 0.09(0.07-0.12) | 0.31(0.22-0.44) | 6.50(4.18-10.11) | 2.93(1.64-5.25) | 1.48(0.74-2.95) | 1.55(0.73-3.30) | 14.67(10.22-21.03) | 3.06(1.93-4.87) |
| Self-efficiency |  |  |  |  |  |  |  |  |
| Level 1 | 1.00(ref.) | 1.00(ref.) | 1.00(ref.) | 1.00(ref.) | 1.00(ref.) | 1.00(ref.) | 1.00(ref.) | 1.00(ref.) |
| Level 2 | 1.56(0.99-0.45) | 0.98(0.61-1.59) | 1.01(0.53-1.94) | 1.31(0.67-2.57) | 1.66(0.51-5.44) | 1.77(0.54-5.83) | 0.43(0.22-0.82) | 0.73(0.37-1.45) |
| Level 3 | 9.68(7.14-13.12) | 3.41(2.42-4.82) | 0.18(0.11-0.28) | 0.34(0.20-0.58) | 0.48(0.24-0.98) | 0.52(0.25-1.08) | 0.06(0.04-0.10) | 0.23(0.13-0.38) |
| Trust in medical staff |  |  |  |  |  |  |  |  |
| Level 1 | 1.00(ref.) | 1.00(ref.) | 1.00(ref.) | 1.00(ref.) | 1.00(ref.) | 1.00(ref.) | 1.00(ref.) | 1.00(ref.) |
| Level 2 | 1.98(1.62-2.43) | 1.32(1.05-1.65) | 0.65(0.47-0.91) | 0.90(0.63-1.29) | 1.31(0.63-2.71) | 1.37(0.66-2.84) | 0.45(0.35-0.58) | 0.72(0.55-0.96) |
| Level 3 | 3.33(2.71-4.11) | 1.54(1.21-1.97) | 0.48(0.35-0.67) | 0.92(0.63-1.34) | 1.09(0.54-2.19) | 1.12(0.55-2.30) | 0.23(0.18-0.31) | 0.58(0.42-0.80) |
| Level 4 | 7.80(5.84-10.41) | 0.95(0.63-1.44) | 0.24(0.15-0.37) | 1.38(0.73-2.62) | 0.62(0.27-1.45) | 0.68(0.28-1.62) | 0.09(0.06-0.13) | 1.04(0.59-1.84) |
| Trust in developers |  |  |  |  |  |  |  |  |
| Level 1 | 1.00(ref.) | 1.00(ref.) | 1.00(ref.) | 1.00(ref.) | 1.00(ref.) | 1.00(ref.) | 1.00(ref.) | 1.00(ref.) |
| Level 2 | 2.95(2.45-3.54) | 1.82(1.47-2.26) | 0.50(0.37-0.67) | 0.65(0.47-0.91) | 0.68(0.32-1.43) | 0.74(0.35-1.59) | 0.31(0.25-0.39) | 0.58(0.44-0.75) |
| Level 3 | 4.27(3.31-5.51) | 1.31(0.96-1.79) | 0.35(0.24-0.53) | 0.72(0.44-1.17) | 0.98(0.44-2.18) | 1.05(0.46-2.39) | 0.19(0.14-0.27) | 0.75(0.50-1.12) |
| Level 4 | 8.73(6.55-11.63) | 0.95(0.63-1.44) | 0.17(0.10-0.27) | 0.62(0.32-1.20) | 1.13(0.55-2.31) | 1.34(0.63-2.85) | 0.08(0.05-0.12) | 1.04(0.59-1.81) |

OR, odds ratio; CI, confidence interval.

Levels 1-4: indicate progressively higher degrees. The higher the degree, the higher level of social status in China/community, the better the self-assessment of health status, the more awareness of COVID-19 vaccine, the more severe/barriers, the greater the susceptibility/benefits, the higher the self-efficacy and the more trust in medical staff and developers.

^a^ unadjusted;

^b^ adjusted age, gender, educational status, subjective social status in China/community, self-report health condition (EQ-5D), chronic disease, the history of allergic, drinking status, physical activity, public health prevention measures, severity, susceptibility, benefits, barriers, self-efficiency, the trust in medical staff, and the trust in developers.

^c^ adjusted gender, educational status, subjective social status in community, self-report health condition (EQ-5D), the history of allergic, physical activity, public health prevention measures, severity, susceptibility, benefits, barriers, self-efficiency, the trust in medical staff, and the trust in developers.

^d^ adjusted chronic disease, physical activity, awareness of COVID-19 vaccines, susceptibility.

^e^ adjusted age, educational status, subjective social status in China/community, self-report health condition (EQ-5D), chronic disease, the history of allergic, drinking status, physical activity, public health prevention measures, awareness of COVID-19 vaccines, severity, susceptibility, benefits, barriers, self-efficiency, the trust in medical staff, and the trust in developers.
