# Supplementary material for: Dynamic Trends and Underlying Factors of COVID-19 Vaccine Booster Hesitancy in Adults: Cross-Sectional Observational Study
Source: JMIR Public Health Surveill. 2023 Aug 1;9:e44822. doi: 10.2196/44822 (PMC10395646; doi:10.2196/44822)
Supplement: Multimedia Appendix 5 [file publichealth_v9i1e44822_app5.docx]

| **Covariates** | **Vaccine hesitancy in booster vaccination** | | | | **Vaccine hesitancy in regular booster vaccination** | | | |
| --- | --- | --- | --- | --- | --- | --- | --- | --- |
|  | **OR(95%CI)^a^** | ***P-*value** | **OR(95%CI)^b^** | ***P-*value** | **OR(95%CI)^a^** | ***P-*value** | **OR(95%CI)^c^** | ***P-*value** |
| Age, years | | | | | | | | |
| 18-29 | 1.00(ref.) |  | 1.00(ref.) |  | 1.00(ref.) |  | 1.00(ref.) |  |
| 30-39 | 1.25(0.93-1.67) | 0.14 | 1.13(0.83-1.55) | 0.44 | 1.19(0.92-1.53) | 0.18 | 1.08(0.82-1.43) | 0.57 |
| 40-49 | 0.78(0.56-1.09) | 0.15 | 0.82(0.57-1.18) | 0.28 | 0.88(0.67-1.17) | 0.39 | 0.98(0.72-1.34) | 0.90 |
| 50-59 | 0.75(0.54-1.04) | 0.09 | 0.82(0.55-1.22) | 0.32 | 0.72(0.54-0.96) | 0.02 | 0.87(0.62-1.24) | 0.45 |
| 60- | 1.23(0.88-1.72) | 0.23 | 1.21(0.79-1.84) | 0.38 | 1.20(0.90-1.60) | 0.21 | 1.39(0.97-2.01) | 0.08 |
| Gender | | | | | | | | |
| Male | 1.00(ref.) |  | 1.00(ref.) |  | 1.00(ref.) |  | 1.00(ref.) |  |
| Female | 1.10(0.92-1.33) | 0.30 | 1.15(0.94-1.40) | 0.18 | 1.20(1.02-1.41) | 0.03 | 1.32(1.07-1.63) | 0.009 |
| Ethnic groups | | | | | | | | |
| Han | 1.00(ref.) |  | 1.00(ref.) |  | 1.00(ref.) |  | 1.00(ref.) |  |
| Minority | 0.87(0.48-1.58) | 0.66 | 0.80(0.42-1.50) | 0.49 | 0.82(0.49-1.38) | 0.46 | 0.76(0.43-1.35) | 0.35 |
| Religion | | | | | | | | |
| Atheist | 1.00(ref.) |  | 1.00(ref.) |  | 1.00(ref.) |  | 1.00(ref.) |  |
| Others | 0.97(0.62-1.53) | 0.90 | 0.97(0.60-1.57) | 0.90 | 1.07(0.73-1.56) | 0.74 | 1.13(0.75-1.70) | 0.56 |
| Marital status | | | | | | | | |
| Married | 1.00(ref.) |  | 1.00(ref.) |  | 1.00(ref.) |  | 1.00(ref.) |  |
| Others | 0.86(0.64-1.15) | 0.32 | 0.75(0.54-1.05) | 0.10 | 0.94(0.74-1.20) | 0.62 | 0.86(0.64-1.14) | 0.29 |
| Educational status | | | | | | | | |
| Below high school | 1.00(ref.) |  | 1.00(ref.) |  | 1.00(ref.) |  | 1.00(ref.) |  |
| High school graduate | 1.38(1.09-1.77) | 0.009 | 1.49(1.14-1.94) | 0.004 | 1.62(1.31-1.99) | <.001 | 1.78(1.41-2.23) | <.001 |
| University graduate | 1.70(1.36-2.11) | <.001 | 1.97(1.48-2.61) | <.001 | 1.92(1.58-2.32) | <.001 | 2.24(1.81-2.78) | <.001 |
| Subjective social status in China | | | | | | | | |
| Level 1 | 1.00(ref.) |  | 1.00(ref.) |  | 1.00(ref.) |  | 1.00(ref.) |  |
| Level 2 | 1.23(0.98-1.53) | 0.07 | 1.18(0.89-1.58) | 0.25 | 1.33(1.10-162) | 0.004 | 1.24(0.96-1.59) | 0.10 |
| Level 3 | 1.00(0.72-1.38) | 0.99 | 0.90(0.56-1.46) | 0.68 | 1.26(0.96-1.65) | 0.09 | 1.10(0.73-1.65) | 0.66 |
| Level 4 | 0.75(0.55-1.03) | 0.08 | 0.90(0.50-1.61) | 0.71 | 0.89(0.68-1.17) | 0.41 | 0.92(0.56-1.52) | 0.75 |
| Subjective social status in Community | | | | | | | | |
| Level 1 | 1.00(ref.) |  | 1.00(ref.) |  | 1.00(ref.) |  | 1.00(ref.) |  |
| Level 2 | 1.23(0.98-1.55) | 0.07 | 1.21(0.90-1.64) | 0.21 | 1.41(1.15-1.73) | 0.001 | 1.36(1.04-1.77) | 0.02 |
| Level 3 | 0.95(0.69-1.32) | 0.78 | 0.98(0.61-1.58) | 0.94 | 1.31(0.99-1.72) | 0.06 | 1.34(0.89-2.01) | 0.16 |
| Level 4 | 0.81(0.60-1.11) | 0.19 | 1.10(0.61-1.97) | 0.76 | 0.98(0.75-1.28) | 0.89 | 1.19(0.72-1.97) | 0.50 |
| Self-report health condition (EQ-5D) | | | | | | | | |
| Level 1 | 1.00(ref.) |  | 1.00(ref.) |  | 1.00(ref.) |  | 1.00(ref.) |  |
| Level 2 | 0.62(0.49-0.78) | <.001 | 0.79(0.61-1.02) | 0.07 | 0.65(0.52-0.80) | <.001 | 0.80(0.63-1.01) | 0.06 |
| Level 3 | 0.43(0.34-0.56) | <.001 | 0.63(0.47-0.83) | 0.001 | 0.56(0.45-0.69) | <.001 | 0.78(0.62-0.99) | 0.04 |
| Level 4 | 0.39(0.30-0.51) | <.001 | 0.66(0.49-0.89) | 0.007 | 0.40(0.32-0.51) | <.001 | 0.64(0.49-0.84) | 0.001 |
| Chronic disease | | | | | | | | |
| Yes | 1.00(ref.) |  | 1.00(ref.) |  | 1.00(ref.) |  | 1.00(ref.) |  |
| No | 0.47(0.37-0.58) | <.001 | 0.49(0.38-0.64) | <.001 | 0.55(0.45-0.68) | <.001 | 0.58(0.45-0.74) | <.001 |
| The history of allergic | | | | | | | | |
| Yes | 1.00(ref.) |  | 1.00(ref.) |  | 1.00(ref.) |  | 1.00(ref.) |  |
| No | 0.33(0.25-0.43) | <.001 | 0.45(0.33-0.61) | <.001 | 0.36(0.28-0.46) | <.001 | 0.49(0.37-0.65) | <.001 |
| Unclear | 0.68(0.49-0.95) | 0.03 | 0.66(0.45-0.95) | 0.03 | 0.70(0.51-0.94) | 0.02 | 0.68(0.49-0.96) | 0.03 |
| Smoking status | | | | | | | | |
| Current smoker | 1.00(ref.) |  | 1.00(ref.) |  | 1.00(ref.) |  | 1.00(ref.) |  |
| Former smoker | 1.12(0.73-1.71) | 0.61 | 1.06(0.67-1.67) | 0.81 | 1.25(0.86-1.81) | 0.25 | 1.21(0.81-1.82) | 0.36 |
| Never smoker | 0.97(0.78-1.22) | 0.82 | 1.00(0.78-1.28) | 0.99 | 1.16(0.95-1.42) | 0.15 | 1.03(0.76-1.39) | 0.85 |
| Drinking status | | | | | | | | |
| Current drinker | 1.00(ref.) |  | 1.00(ref.) |  | 1.00(ref.) |  | 1.00(ref.) |  |
| Former drinker | 1.41(0.95-2.08) | 0.09 | 1.26(0.83-1.93) | 0.28 | 1.42(1.01-1.99) | 0.046 | 1.29(0.88-1.87) | 0.19 |
| Never drinker | 0.97-0.78-1.20) | 0.78 | 1.09(0.86-1.37) | 0.49 | 1.01(0.84-1.21) | 0.92 | 0.92(0.73-1.17) | 0.52 |
| Physical activity | | | | | | | | |
| High level | 1.00(ref.) |  | 1.00(ref.) |  | 1.00(ref.) |  | 1.00(ref.) |  |
| Middle level | 1.54(1.25-1.91) | <.001 | 1.18(0.93-1.48) | 0.17 | 1.49(1.24-1.78) | <.001 | 1.18(0.97-1.44) | 0.11 |
| Low level | 1.98(1.56-2.52) | <.001 | 1.34(1.03-1.74) | 0.03 | 1.86(1.51-2.29) | <.001 | 1.28(1.01-1.61) | 0.04 |
| Public health prevention measures | | | | | | | | |
| Low level | 1.00(ref.) |  | 1.00(ref.) |  | 1.00(ref.) |  | 1.00(ref.) |  |
| Middle level | 0.86(0.60-1.23) | 0.41 | 0.79(0.54-1.17) | 0.24 | 1.06(0.77-1.47) | 0.71 | 1.01(0.71-1.44) | 0.94 |
| High level | 0.38(0.29-0.50) | <.001 | 0.58(0.43-0.79) | <.001 | 0.44(0.34-0.57) | <.001 | 0.65(0.50-0.86) | 0.002 |
| Awareness of COVID-19 vaccines | | | | | | | | |
| Level 1 | 1.00(ref.) |  | 1.00(ref.) |  | 1.00(ref.) |  | 1.00(ref.) |  |
| Level 2 | 0.69(0.51-0.94) | 0.02 | 0.78(0.56-1.08) | 0.14 | 0.82(0.63-1.06) | 0.13 | 0.93(0.70-1.24) | 0.62 |
| Level 3 | 0.81(0.64-1.03) | 0.09 | 1.04(0.80-1.35) | 0.75 | 0.99(0.80-1.21) | 0.89 | 1.31(1.05-1.65) | 0.02 |
| Level 4 | 0.72(0.57-0.92) | 0.009 | 0.85(0.65-1.12) | 0.25 | 0.91(0.74-1.12) | 0.38 | 1.12(0.89-1.41) | 0.34 |
| Channel of vaccine information | | | | | | | | |
| We Media | 1.00(ref.) |  | 1.00(ref.) |  | 1.00(ref.) |  | 1.00(ref.) |  |
| Official media | 0.84(0.60-1.17) | 0.30 | 1.03(0.72-1.47) | 0.89 | 0.83(0.62-1.10) | 0.20 | 1.03(0.75-1.41) | 0.88 |
| Others | 0.78(0.64-0.96) | 0.02 | 1.00(0.80-1.24) | 0.99 | 0.81(0.68-0.97) | 0.02 | 1.04(0.86-1.26) | 0.68 |
| Severity | | | | | | | | |
| Level 1 | 1.00(ref.) |  | 1.00(ref.) |  | 1.00(ref.) |  | 1.00(ref.) |  |
| Level 2 | 1.77(1.37-2.30) | <.001 | 0.91(0.68-1.22) | 0.54 | 1.77(1.42-2.21) | <.001 | 0.92(0.71-1.18) | 0.51 |
| Level 3 | 1.58(1.22-2.03) | <.001 | 1.07(0.81-1.43) | 0.62 | 1.53(1.24-1.90) | <.001 | 1.03(0.80-1.32) | 0.82 |
| Level 4 | 0.84(0.60-1.17) | 0.30 | 1.80(1.20-2.69) | 0.004 | 0.81(0.61-1.08) | 0.15 | 1.98(1.38-2.82) | <.001 |
| Susceptibility | | | | | | | | |
| Level 1 | 1.00(ref.) |  | 1.00(ref.) |  | 1.00(ref.) |  | 1.00(ref.) |  |
| Level 2 | 1.17(0.90-1.52) | 0.23 | 0.49(0.37-0.66) | <.001 | 1.29(1.03-1.61) | 0.03 | 0.52(0.40-0.67) | <.001 |
| Level 3 | 1.42(1.11-1.82) | 0.005 | 0.37(0.28-0.50) | <.001 | 1.59(1.28-1.97) | <.001 | 0.41(0.32-0.53) | <.001 |
| Level 4 | 1.06(0.77-1.46) | 0.72 | 0.43(0.30-0.62) | <.001 | 1.11(0.84-1.47) | 0.45 | 0.43(0.31-0.60) | <.001 |
| Benefits | | | | | | | | |
| Level 1 | 1.00(ref.) |  | 1.00(ref.) |  | 1.00(ref.) |  | 1.00(ref.) |  |
| Level 2 | 0.34(0.27-0.42) | <.001 | 0.50(0.40-0.63) | <.001 | 0.38(0.32-0.45) | <.001 | 0.54(0.44-0.66) | <.001 |
| Level 3 | 0.17(0.13-0.23) | <.001 | 1.04(0.72-1.52) | 0.82 | 0.17(0.13-0.21) | <.001 | 1.03(0.74-1.42) | 0.87 |
| Barriers | | | | | | | | |
| Level 1 | 1.00(ref.) |  | 1.00(ref.) |  | 1.00(ref.) |  | 1.00(ref.) |  |
| Level 2 | 1.66(1.16-2.38) | 0.006 | 0.72(0.48-1.09) | 0.12 | 2.35(1.71-3.23) | <.001 | 0.95(0.66-1.37) | 0.79 |
| Level 3 | 4.69(3.20-6.86) | <.001 | 1.54(0.99-2.42) | 0.06 | 6.83(4.88-9.56) | <.001 | 2.13(1.44-3.16) | <.001 |
| Level 4 | 10.23(7.42-14.10) | <.001 | 3.16(2.05-4.86) | <.001 | 12.44(9.25-16.72) | <.001 | 3.63(2.46-5.34) | <.001 |
| Self-efficiency | | | | | | | | |
| Level 1 | 1.00(ref.) |  | 1.00(ref.) |  | 1.00(ref.) |  | 1.00(ref.) |  |
| Level 2 | 0.57(0.32-1.04) | 0.07 | 0.87(0.47-1.63) | 0.67 | 0.64(0.39-1.04) | 0.07 | 0.96(0.57-1.62) | 0.89 |
| Level 3 | 0.11(0.07-0.16) | <.001 | 0.32(0.21-0.50) | <.001 | 0.10(0.07-0.14) | <.001 | 0.28(0.19-0.42) | <.001 |
| Trust in medical staff | | | | | | | | |
| Level 1 | 1.00(ref.) |  | 1.00(ref.) |  | 1.00(ref.) |  | 1.00(ref.) |  |
| Level 2 | 0.43(0.33-0.55) | <.001 | 0.67(0.51-0.88) | 0.004 | 0.46(0.37-0.57) | <.001 | 0.75(0.59-0.95) | 0.02 |
| Level 3 | 0.26(0.20-0.34) | <.001 | 0.55(0.40-0.75) | <.001 | 0.29(0.23-0.36) | <.001 | 0.68(0.52-0.89) | 0.005 |
| Level 4 | 0.11(0.08-0.17) | <.001 | 0.79(0.46-1.33) | 0.37 | 0.12(0.09-0.17) | <.001 | 1.26(0.81-1.98) | 0.31 |
| Trust in developers | | | | | | | | |
| Level 1 | 1.00(ref.) |  | 1.00(ref.) |  | 1.00(ref.) |  | 1.00(ref.) |  |
| Level 2 | 0.29(0.23-0.37) | <.001 | 0.56(0.43-0.73) | <.001 | 0.32(0.27-0.39) | <.001 | 0.55(0.44-0.69) | <.001 |
| Level 3 | 0.22(0.16-0.31) | <.001 | 0.87(0.60-1.27) | 0.48 | 0.21(0.16-0.27) | <.001 | 0.65(0.47-0.91) | 0.01 |
| Level 4 | 0.13(0.09-0.18) | <.001 | 1.56(0.95-2.58) | 0.08 | 0.09(0.07-0.13) | <.001 | 0.80(0.51-1.26) | 0.33 |

OR, odds ratio; CI, confidence interval.

Levels 1-4: indicate progressively higher degrees. The higher the degree, the higher level of social status in China/community, the better the self-assessment of health status, the more awareness of COVID-19 vaccine, the more severe/barriers, the greater the susceptibility/benefits, the higher the self-efficacy and the more trust in medical staff and developers.

^a^ unadjusted;

^b^ adjusted age, educational status, subjective social status in China/community, self-report health condition (EQ-5D), chronic disease, the history of allergic, drinking status, physical activity, public health prevention measures, awareness of COVID-19 vaccines, severity, susceptibility, benefits, barriers, self-efficiency, the trust in medical staff, and the trust in developers.

^c^ adjusted age, gender, educational status, subjective social status in China/community, self-report health condition (EQ-5D), chronic disease, the history of allergic, drinking status, physical activity, public health prevention measures, severity, susceptibility, benefits, barriers, self-efficiency, the trust in medical staff, and the trust in developers.
